# Small Open Reading Frames, Non-Coding RNAs and Repetitive Elements in *Bradyrhizobium japonicum* USDA 110

**DOI:** 10.1371/journal.pone.0165429

**Published:** 2016-10-27

**Authors:** Julia Hahn, Olga V. Tsoy, Sebastian Thalmann, Jelena Čuklina, Mikhail S. Gelfand, Elena Evguenieva-Hackenberg

**Affiliations:** 1 Institute of Microbiology and Molecular Biology, Justus-Liebig-University, Giessen, Germany; 2 A. A. Kharkevich Institute for Information Transmission Problems, Russian Academy of Sciences, Bolshoi Karetny Ln. 19, Moscow, 127051, Russia; 3 ETH, Institute of Molecular Systems Biology, Zürich, Switzerland; 4 Skolkovo Institute of Science and Technology, Nobel Str. 3, Moscow, 143026, Russia; 5 Faculty of Bioengineering and Bioinformatics, M. V. Lomonosov Moscow State University, Vorobyevy Gory 1–73, Moscow, 119234, Russia; 6 Faculty of Computer Science, Higher School of Economics, Kochnovsky Dr. 3, Moscow, 125319, Russia; INSERM U869, FRANCE

## Abstract

Small open reading frames (sORFs) and genes for non-coding RNAs are poorly investigated components of most genomes. Our analysis of 1391 ORFs recently annotated in the soybean symbiont *Bradyrhizobium japonicum* USDA 110 revealed that 78% of them contain less than 80 codons. Twenty-one of these sORFs are conserved in or outside Alphaproteobacteria and most of them are similar to genes found in transposable elements, in line with their broad distribution. Stabilizing selection was demonstrated for sORFs with proteomic evidence and bll1319_ISGA which is conserved at the nucleotide level in 16 alphaproteobacterial species, 79 species from other taxa and 49 other Proteobacteria. Further we used Northern blot hybridization to validate ten small RNAs (BjsR1 to BjsR10) belonging to new RNA families. We found that BjsR1 and BjsR3 have homologs outside the genus *Bradyrhizobium*, and BjsR5, BjsR6, BjsR7, and BjsR10 have up to four imperfect copies in *Bradyrhizobium* genomes. BjsR8, BjsR9, and BjsR10 are present exclusively in nodules, while the other sRNAs are also expressed in liquid cultures. We also found that the level of BjsR4 decreases after exposure to tellurite and iron, and this down-regulation contributes to survival under high iron conditions. Analysis of additional small RNAs overlapping with 3’-UTRs revealed two new repetitive elements named Br-REP1 and Br-REP2. These REP elements may play roles in the genomic plasticity and gene regulation and could be useful for strain identification by PCR-fingerprinting. Furthermore, we studied two potential toxin genes in the symbiotic island and confirmed toxicity of the *yhaV* homolog bll1687 but not of the newly annotated *higB* homolog blr0229_ISGA in *E*. *coli*. Finally, we revealed transcription interference resulting in an antisense RNA complementary to blr1853, a gene induced in symbiosis. The presented results expand our knowledge on sORFs, non-coding RNAs and repetitive elements in *B*. *japonicum* and related bacteria.

## Introduction

High-throughput transcriptomic and proteomic studies reveal many new genes in bacteria. However, bacterial genes for regulatory small non-coding RNAs (sRNAs) are still poorly characterized [1] and genes for small proteins often remain unrecognized [2–5], although many small proteins perform important structural and regulatory functions (reviewed in refs. [4,5]) or are parts of toxin-antitoxin systems (TAs) that are important for survival of bacterial subpopulations under detrimental conditions [6,7]. Hence, systematic, large-scale identification of these genome elements followed by detailed characterization is an important research area.

*Bradyrhizobium japonicum* USDA 110 (recently re-named to *Bradyrhizobium diazoefficens* USDA 110 [8]) is a slow-growing rhizobium belonging to the Alphaproteobacteria phylum. It lives in soil and can fix molecular nitrogen in symbiosis with the soybean plant hence acting as a natural fertilizer [9,10]. With 9.1 Mb, the genome of *B*. *japonicum* USDA 110 is one of the largest bacterial genomes that have been sequenced in the early period of the genomics research [11]. Early proteomic and microarray analyses of *B*. *japonicum* provided an inventory of its protein coding genes [12–14]. Recently, differential RNA-seq (dRNA-seq) was used for mapping of transcription start sites (TSSs) onto the re-annotated genome of *B*. *japonicum* USDA 110 [15]. This re-annotation added 1391 new putative protein-coding genes (107 of them with proteomic evidence) to the 8317 previously annotated ones [11,15]. Further, thousands of TSSs were mapped to non-annotated regions or in the opposite orientation to genes in *B*. *japonicum*, indicating that they may correspond to *trans*- or *cis*-acting, non-coding RNAs [15]. Virtually all newly annotated ORFs and most putative small RNAs (sRNAs) of *B*. *japonicum* have not been studied yet.

*Trans*-acting bacterial sRNAs are usually transcribed from orphan genes, but they can also be derived from untranslated mRNA regions [16,17], while c*is-*acting sRNAs are transcribed as antisense RNAs (asRNAs) [18]. asRNAs are known to regulate plasmid replication, transposition, and toxin manifestation in TA systems. They can influence stability, translation, and transcription termination of the complementary mRNAs (recently summarized in [19]). However, an asRNA can also be a by-product of transcription interference between closely located promoters and may lack specific function [19]. Most *trans*-acting sRNAs bind specific mRNAs and influence their translation and/or stability. In this way sRNAs regulate, amongst others, adaptation to changing environmental conditions, central metabolism, photosynthesis, quorum sensing and virulence [20–25]. Generally, *trans*-acting sRNAs that interact with mRNAs are not highly conserved: conservation of some regulatory sRNAs in bacterial families was reported [26, 27], but species-specific sRNAs with important functions were also described [23]. Currently, only nine *B*. *japonicum* sRNAs belonging to seven RNA families are known [28,29].

Recently the 3’-UTRs of bacterial mRNAs emerged as a source for *trans*-acting sRNAs [17]. In *B*. *japonicum*, clustering of TSSs near the end of ORFs was observed [15], suggesting widespread existence of sRNAs and asRNAs associated with 3’-UTRs. It is noteworthy that some bacterial 3’-UTRs comprise stem-loop structures resulting from transcription of repetitive extragenic palindromic elements (REPs), which are present in hundreds of copies in bacterial genomes [30–32]. REPs are best studied in *Escherichia coli*, where at RNA levels they can have several functions. REPs impede exoribonucleolytic degradation in 3’-5’ direction and stabilize upstream mRNA coding regions [33], function in transcription termination [34], and, if located within 15 nt of a stop codon, stall ribosome movement and induce mRNA decay in the trans-translation process [35]. The presence of REPs in *B*. *japonicum* 3’-UTRs was not shown so far, although primers corresponding to enterobacterial and streptococcal REPs were used for PCR-fingerprinting of bradyrhizobia [36–38].

The aim of this study was to use the recent re-annotation of the *B*. *japonicum* USDA 110 genome with mapped TSSs in order to identify and characterize sORFs and sRNAs. We found 1080 annotated sORFs and analyzed their phylogenetic distribution. Beyond sORFs showing homology to genes usually present in transposable elements, we found three conserved sORFs with homologs outside Alphaproteobacteria. Further, we identified sRNAs belonging to ten new RNA families, analyzed their expression and conservation, and found that down-regulation of the sRNA BjsR4 probably contributes to survival at high iron concentrations. Furthermore, we analyzed sRNA candidates associated with 3’-UTRs and identified two new repetitive elements, which are widely distributed in the *Bradyrhizobium* and *Rhodopseudomonas* genera. Finally, we validated a toxin gene and detected a case of transcription interference in the symbiotic island. Our results broaden the knowledge on sORFs, non-coding RNAs, and repetitive elements in *B*. *japonicum* and related bacteria.

## Materials and Methods

### Bioinformatic analyses

Conservation of sORFs was analyzed using the Basic Local Alignment Search Tool (BLAST) [39] with the e-value threshold of 10^−5^. For the calculation of the ratio of the number of non-synonymous substitutions to the number of synonymous ones (dN/dS), PAML [40] was used.

The Rfam database was searched for known RNA families [41]. Homologs of *B*. *japonicum* USDA 110 sRNAs were identified by BLASTN [39] and subject to multiple sequence alignments and secondary structure prediction [42]. Phyre2 was used for prediction of secondary and tertiary structures of proteins [43].

### Visualization of dRNA-seq data

Previously published dRNA-seq data [15] were used to visualize cDNA reads corresponding to loci of interest. Visualization was performed with the Integrated Genome Browser [http://bioviz.org/igb/index.html] using the recent re-annotation of the *B*. *japonicum* USDA 110 genome [15].

### Cultivation methods and cloning procedures

*B japonicum* 110*spc*4 [44], a spectinomycin-resistant derivative of *B*. *japonicum* USDA 110, was either grown in liquid cultures in PSY medium [45] with spectinomycin (100 μg ml^-1^) at 30°C. Soybean nodules containing *B*. *japonicum* bacteroides [13] and uninfected soybean roots were kindly provided by Hans-Martin Fischer (ETH Zürich, Switzerland). *Escherichia coli* JM109 was cultivated in LB medium and standard cloning procedures were used [46, 47]. Cloning of putative toxin genes in *E*. *coli* was performed in pBAD_Cm [48]. Suitable constructs for measurement of promoter activities in *B*. *japonicum* were cloned in pME3535XhoI or its derivatives, cleaved out with EcoRI and XhoI and re-cloned into the broad host range plasmid pRK290XhoI [49]. For overexpression of BjsR4 in *B*. *japonicum* we used a modified pRJPaph-gfp_a1 [50] plasmid in which the *gfp* gene was replaced by multiple cloning site resulting in pRJPaph-MCS. The sequence corresponding to BjsR4 was cloned downstream of the constitutive Paph promoter between the KpnI and SpeI restriction sites of pRJPaph-MCS resulting in the plasmid pRJPaph-bjsR4. Plasmids were transferred from *E*. *coli* S17-1 to *B*. *japonicum* 110*spc*4 by biparental conjugation [51]. Used plasmids and oligonucleotides are listed in S1 and S2 Tables, respectively.

### RNA isolation and Northern blot analysis

Total RNA from free-living cells in the exponential growth phase (OD_600_ of 0.4 to 0.6) or stationary growth phase (OD_600_ of 1.2) as well as total RNA from soybean nodules and from uninfected soybean roots was isolated with hot-phenol [13]. RNA separation in urea-containing 10% polyacrylamide gels, semidry blotting, hybridization with radioactively labelled oligonucleotide probes, re-hybridization and signal detection were performed as described [52]. Oligonucleotides used for hybridization are listed in S2 Table.

## Results and Discussion

### Conservation of *B*. *japonicum* small ORFs

**Fig 1:**
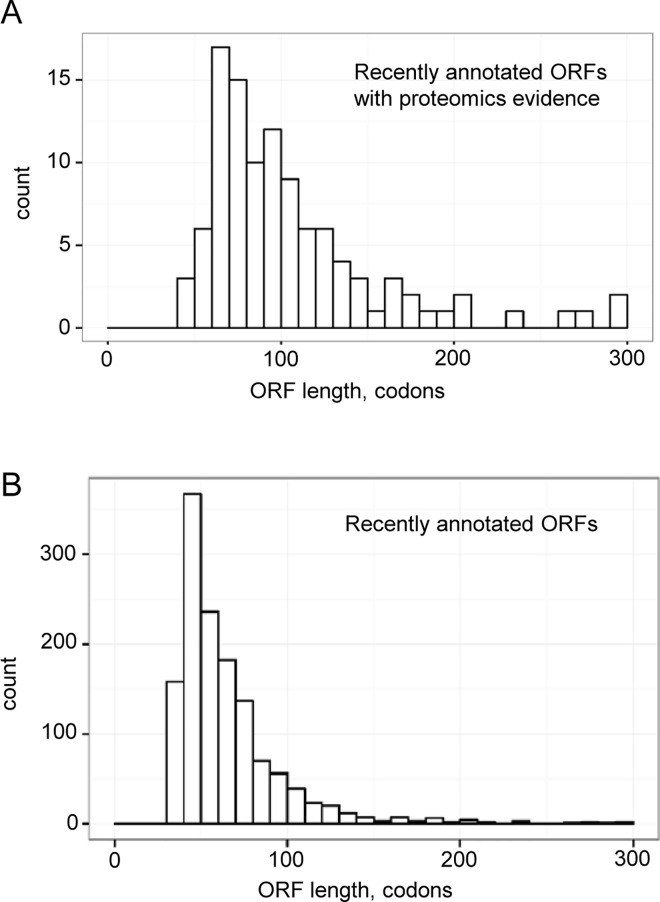
Length distribution of the recently annotated ORFs of *B*. *japonicum* USDA 110. **A)** Length distribution of 107 ORFs with proteomic evidence. **B**) Length distribution of all 1391 new ORFs.

Assuming that small genes are underrepresented in the original RefSeq annotation of the *B*. *japonicum* USDA 110 genome [11] and have been found among the recently annotated ones [15], we analyzed the length distribution of the new genes (Fig 1). For 107 new genes with proteomics support [15] we found a peak corresponding to ORFs with the length between 60 to 80 codons (Fig 1A). Out of these 107 genes, 39 were shorter than 80 amino acids (aa). Then we analyzed the length distribution of all 1391 recently annotated genes and found a clear peak for ORFs containing 40 to 50 codons (Fig 1B). More than 50% of the newly annotated but only three of the experimentally identified new proteins are shorter than 50 aa (compare Fig 1A to Fig 1B). Considering the length distribution of new genes with proteomic evidence, we decided to include in our computational analysis only ORFs with the maximal length of 80 aa (designated sORFs in this work). They represent 78% of the newly annotated genes or 1080 sORFs.

**Fig 2:**
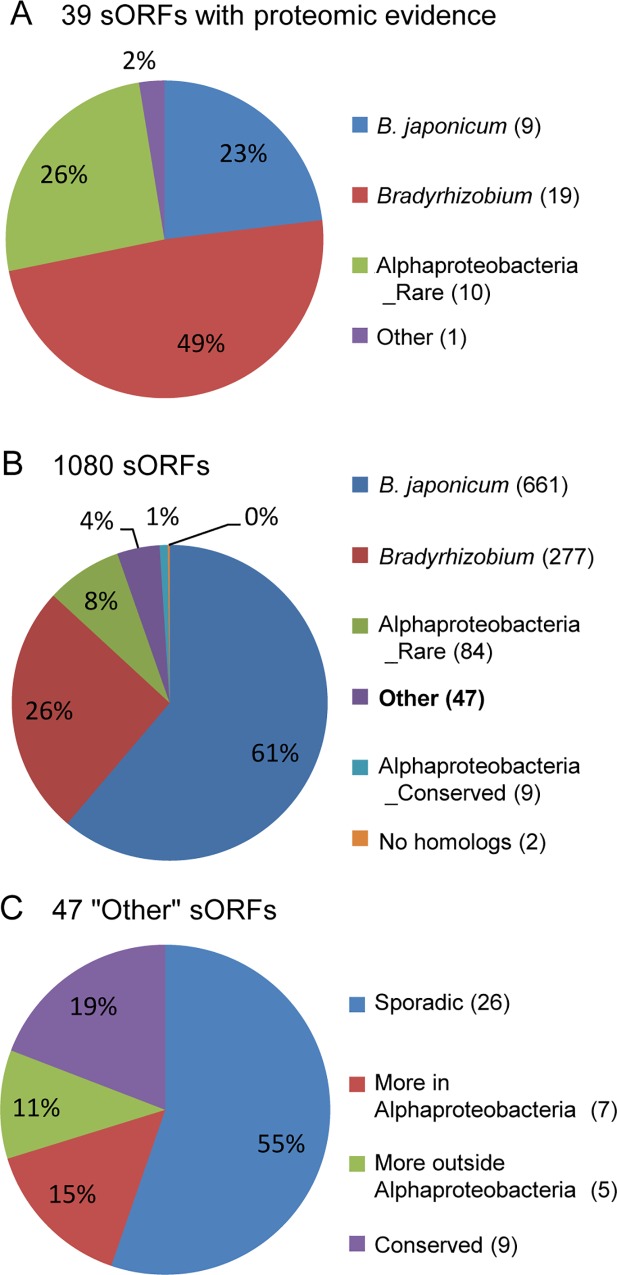
TBLASTX analysis of *B*. *japonicum* sORFs showing the distribution of homologs among bacteria. **A)** Analysis of 39 sORFs with proteomic evidence. Indicated is their presence only in *B*. *japonicum* strains (*B*. *japonicum*), in the genus *Bradyrhizobium*, or in Alphaproteobacteria (S4 Table). Other – the sORF blr0566_ISGA present in Alphaproteobacteria and outside Alphaproteobacteria. **B)** Analysis of all 1080 sORFs (with and without proteomic evidence) (S6 Table). Alphaproteobacteria_Rare – sORFs found in less than five Alphaproteobacteria other than *Bradyrhizobium* spp.; Alphaproteobacteria_Conserved – sORFs found in five or more Alphaproteobacteria other than *Bradyrhizobium* spp.; Other – sORFs found in organisms outside Alphaproteobacteria; No homologs, – sORFs found only in *B*. *japonicum* USDA 110. **C)** Analysis of 47 sORFs with homologs outside Alphaproteobacteria (belonging to the category “Other”). Sporadic – sORFs found in less than 20 Alphaproteobacteria and less than 20 organisms outside Alphaproteobacteria; More in Alphaproteobacteria – sORFs found in at least 20 Alphaproteobacteria and less than 20 organisms outside Alphaproteobacteria; More outside Alphaproteobacteria – sORFs found in less than 20 Alphaproteobacteria and at least 20 organisms outside Alphaproteobacteria; Conserved – sORFs found in at least 20 Alphaproteobacteria and at least 20 organisms outside Alphaproteobacteria (S9 Table).

To identify sORFs conserved in Alphaproteobacteria, we analyzed the newly annotated genes using the BLASTN and TBLASTX programs. Among 39 sORFs with the proteomic evidence, the BLASTN analysis revealed 28 sORFs with homologs in species other than *B*. *japonicum* (S1 Fig, S3 Table), while TBLASTX identified 30 such sORFs (Fig 2A, S4 Table). Among these 28 and 30 sORFs, respectively, both programs found homologs for 16 sORFs in other *Bradyrhizobium* spp. and for five sORFs, in some other Alphaproteobacteria. For eight sORFs TBLASTX identified more distant homologs than BLASTN. For three sORFs, however, a larger number of distant homologs were identified by BLASTN than by TBLASTX, but each of these distant homologs was found only in one organism (bll0972_ISGA and blr1194_ISGA with homologs in *Bradyrhizobium* sp. S23321 and bll0720_ISGA with a homolog in *Nitrobacter hamburgensis* X14; see S3 and S4 Tables).

**Fig 3:**
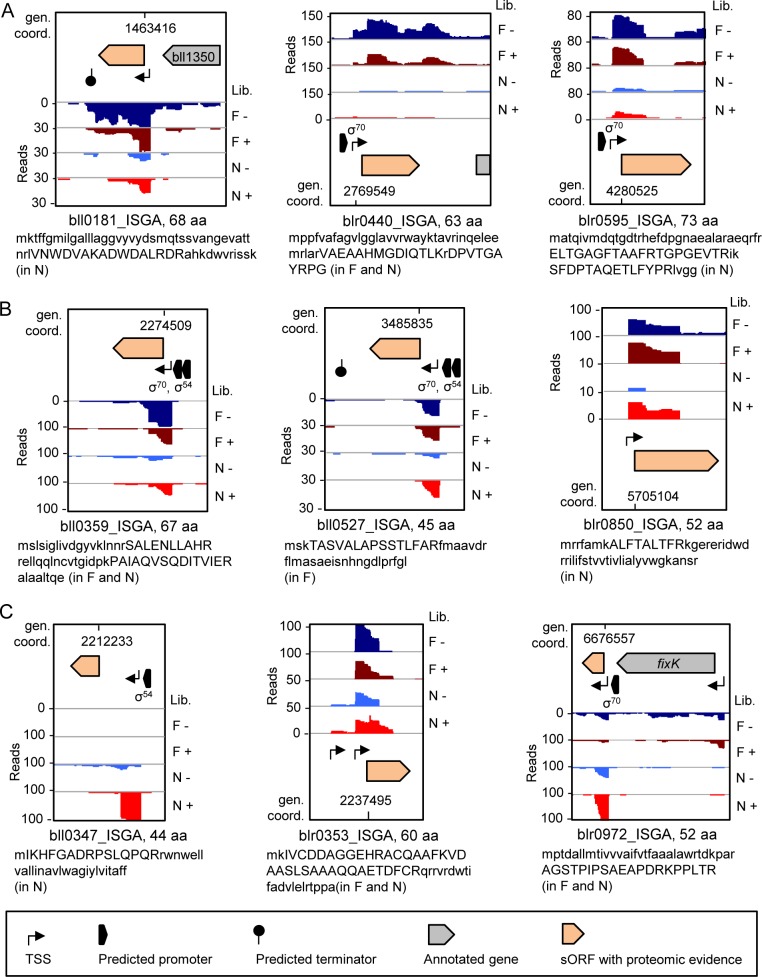
**Expression of sORFs belonging to the groups Alphaproteobacteria_Rare (A), *Bradyrhizobium* (B) and *B*. *japonicum* (C) at RNA and protein level.** The groups are described in Fig 2A. In each panel cDNA reads at the sORF loci are shown [15]. RNA was isolated from exponentially growing, free-living cells (F) in liquid cultures and from nodules (N). RNA samples were treated (+) or not treated (–) with terminal exonuclease (TEX), which degrades 5’-monophosphorylated (processed) transcripts. The scale of each library (Lib.) is indicated (Reads). Annotated features (TSSs, promoters, terminators and closely located flanking genes) are as in ref. [15]. For flanking genes of all shown sORFs, see S4 Table. σ^70^or σ^54^ near the promoter symbol indicates RpoD-like or RpoN-like putative promoter. The genomic coordinates of the sORFs start are given (gen. coord.) Below each panel, the amino acid sequence of the corresponding small proteins is shown. Detected peptides are in uppercase letters; detection of the peptides in Nodules (N) and/or in Free-living condition (F) is indicated in parentheses near the protein sequence. For more details on the proteomic evidence, see Additional File 7: S5 Table in ref. [15].

Only one of 39 sORFs with proteomic evidence (blr0566_ISGA) has homologs outside Alphaproteobacteria (S4 Table). It overlaps with blr3688, a gene not supported by proteomic analysis; both genes lack functional annotation. At the RNA level, this locus is weakly expressed in the liquid culture and in nodules, and no TSS is located close to the sORF (S2 Fig, ref. [15]). In contrast, most sORFs with proteomic evidence have own TSSs and even predicted promoters (S4 Table and Fig 3).

The analysis of all 1080 sORFs revealed 20 sORFs with homologs outside Alphaproteobacteria by BLASTN (S1 Fig, S5 Table) and 47 sORFs with putative homologs outside Alphaproteobacteria by TBLASTX (Fig 2B, S6 Table). Twelve sORFs overlap between these two sets, for eight sORFs only BLASTN found homologs outside Alphaproteobacteria, and for 35 sORFs distant homology was identified only by TBLASTX (S5 and S6 Tables).

A more detailed analysis of 20 sORFs with homologs outside Alphaproteobacteria, which were found by BLASTN, identified the bll1319_ISGA gene conserved at the nucleotide level in 16 alphaproteobacterial species and in 79 species from other taxa, 49 other Proteobacteria (mainly *Pseudomonas*) and at least 30 Actinobacteria (S1 Fig; S7 and S8 Tables).

Forty seven sORFs with putative homologs outside Alphaproteobacteria, which were found by TBLASTX, were also analyzed in more detail (Fig 2C). Twenty six sORFs were distributed sporadically both in and outside Alphaproteobacteria and 21 sORFs had many homologs in and/or outside Alphaproteobacteria: seven sORFs had numerous homologs in Alphaproteobacteria and were distributed sporadically outside this taxon; five sORFs, vice versa, were found in many organisms outside Alphaproteobacteria and only rarely in Alphaproteobacteria; and nine sORFs (including previously identified bll1319_ISGA) were conserved in many organisms at the amino acid level both in and outside Alphaproteobacteria (S9 Table).

Next we analyzed 21 sORFs that had many homologs in and/or outside Alphaproteobacteria. This analysis has shown that twelve of these 21 sORFs resemble genes involved in the maintenance of transposable elements (S10 Table) such as putative transposases, a resolvase and a reverse transcriptase. Seven sORFs overlap with conserved ORFs with already known functions, one of them being a 3’-fragment of a longer conserved gene (S10 Table). The two remaining candidates (blr0250_ISGA and bll1319_ISGA) are homologous to the C-terminal parts of bacterial dTDP-4-dehydrorhamnose 3,5-epimerase and succinate-semialdehyde dehydrogenase, respectively. At the RNA-level, the expression of blr0250_ISGA is higher under symbiosis than during semi-aerobic growth in the liquid culture (S3 Fig), while bll1319_ISGA is not expressed under these conditions (S4 Fig) [15]. Organisms harboring putative bll1319_ISGA homologs and blr0250_ISGA homologs identified using TBLASTX are listed in S8 and S11 Tables, respectively.

The broad conservation of the sORFs bll1319_ISGA and blr0250_ISGA among other bacteria suggests functionality. Their sequences and the sequence of the most conserved sORF with proteomic evidence (blr0566) are shown in S2–S4 Figs, respectively, along with preceding putative Shine-Dalgarno sequences, corresponding protein sequences and predicted secondary and tertiary structures of the proteins. The roles of these conserved sORFs remain to be elucidated.

In summary, our analysis of recently annotated ORFs shows that most of them are small. The observation that most of the newly annotated sORFs but only few of the sORFs with proteomic evidence have less than 50 codons could be explained with experimental difficulties to detect small proteins, but may also indicate annotation of spurious ORFs. The detailed analysis of 21 conserved sORFs found by TBLASTX in and/or outside Alphaproteobacteria showing that most of them are similar to genes of transposable elements suggests that their broad distribution is due to horizontal gene transfer (HGT). HGT can also be assumed for the distribution of putative sORFs found sporadically in some taxa (for example, 26 sORFs found sporadically in and outside Alphaproteobacteria, Fig 2C and S9 Table).

### Stabilizing selection for sORFs

**Fig 4:**
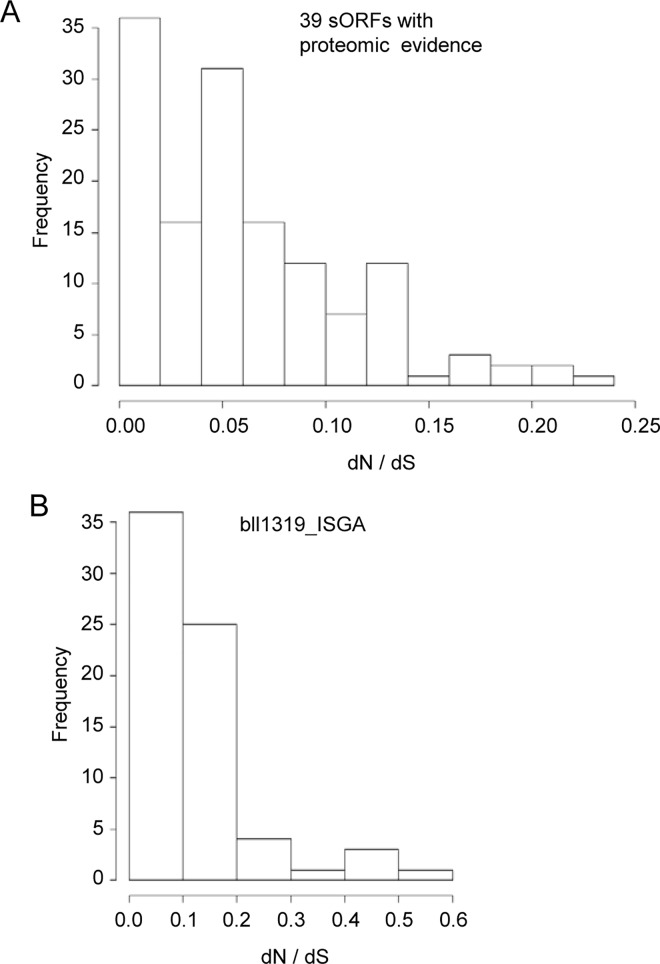
Stabilizing selection for sORFs with proteomic evidence and bll1319_ISGA. **A)** Distribution of the dN/dS ratio for homologs of 39 sORFs with proteomic evidence. **B)** Distribution of the dN/dS ratio for othologs of bll1319_ISGA. All pairs of homologs were considered to construct the histograms.

To confirm functionality of the predicted sORFs, we determined the pattern of selection for sORFs with the proteomic evidence and the bll1319_ISGA gene conserved in many species both at the nucleotide and the amino acid levels. We calculated the ratio of the rate of non-synonymous substitutions to the rate of synonymous ones (dN/dS) (see Methods). Fig 4A represents the histogram of the dN/dS ratios for pairwise alignments of 28 sORFs with proteomic evidence and homologs outside *B*. *japonicum* identified by BLASTN (Fig 2A and S3 Table), whereas Fig 4B features the histogram for bll1319_ISGA. In both cases the ratios are much less than one, demonstrating stabilizing selection. This suggests that bll1319_ISGA and other sORFs conserved at the nucleotide level may encode functional proteins.

### New sRNAs in *B*. *japonicum*

**Fig 5:**
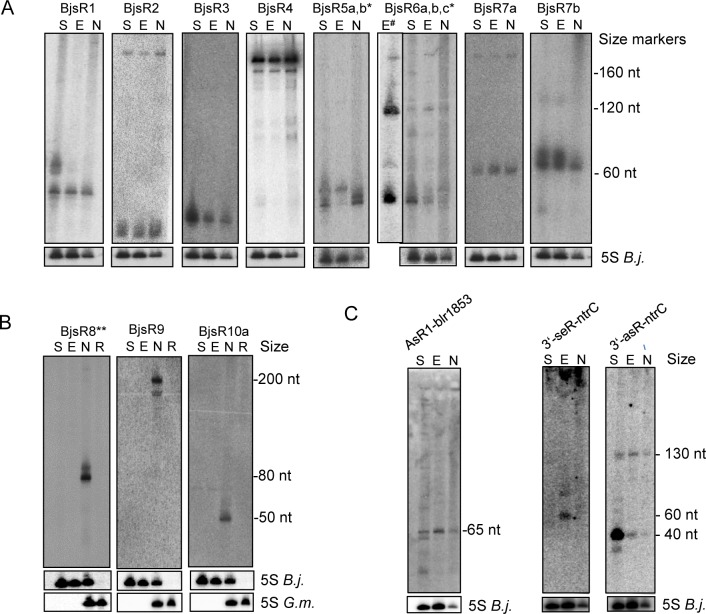
Non-annotated transcripts analyzed by Northern blot hybridization. **A)** New sRNAs detected in liquid cultures and in nodules. **B)** New sRNAs detected only in nodules. **C)** mRNA-associated small transcripts with previously verified TSSs [15]. Total RNA from the exponential growth phase (E) and the stationary phase (S) of a liquid culture and from nodules (N) was used. For sRNAs detected in N only, control RNA from roots (R) was also included. After hybridization with sRNA-specific probes (indicated above the panels), the membranes were re-hybridized with probes specific for 5S rRNA from *B*. *japonicum* (5S *B*.*j*.) and, when root RNA was included, from *G*. *max* (5S *G*.*m*). In A) and B), the positions of the marker RNAs on the membranes (160 nt, 120 nt and 60 nt corresponding to 6S rRNA, 5S rRNA and a fragment detected by the 6S RNA-specific probe, respectively [29]) are given on the right side (in nt). In C), the approximate lengths of the indicated bands are given (in nt), as calculated from the migration of the marker RNAs mentioned above. BjsR5a,b* and BjsR6a,b,c* – no discrimination between homologs in the Northern blot hybridization; BjsR8** – processing product according to dRNA-seq (S12 Fig); E# – RNA isolated by TRIzol resulting in enrichment of sRNAs [53]; all other RNA samples were isolated by hot phenol.

To found new sRNAs which can be validated by Northern blot analysis, we selected nine previously mapped TSSs [15] with high RNA-seq peaks that are located in intergenic regions and probably correspond to orphan sRNAs (Table 1; S5–S11 Figs, S13 and S14 Figs). One additional sRNA candidate without a mapped TSS was included in the analysis because it corresponds to a prominent dRNA-seq peak between *nifH* and *nifQ* (S12 Fig). The ten sRNA candidates (the *B*. *j**aponicum* sRNA candidates BjsR1 to BjsR10) were subjected to Northern blot hybridization using total RNA from nodules and from liquid cultures grown to the exponential and stationary growth phases. The sRNA hybridization patterns are shown in Fig 5A and 5B. Corresponding dRNA-seq data, alignments with homologous sequences of other bacteria, and proposed secondary structures are shown in S5–S14 Figs. As mentioned above, nine validated sRNAs correspond to primary transcripts with mapped TSSs and predicted promoters (Table 1). An interesting exception is BjsR8, which is probably a processing product overlapping with the 3’-UTR of *nifH* mRNA (S12 Fig). Analysis of conservation of the sRNAs in other bacteria revealed that most of them are specific to *Bradyrhizobium* (Table 1). Homologs outside of this genus were found only for BjsR1 and BjsR3 (S5 and S7 Figs). Sequences homologous to the terminator stem-loop of BjsR3 are present in many members of *Rhizobiales* and *Rhodobacterales* including *Brucella*, *Mesorhizobium*, and *Phaeobacter*. Furthermore, we found that BjsR5, BjsR6, BjsR7, and BjsR10 are present in 2 to 4 imperfect copies in *Bradyrhizobium* genomes (S9–S11 and S14 Figs).

**Table 1:** Characteristics of ten sRNAs analyzed by Northern blot hybridization.

sRNA	Strand	TSS or 5‘-end genome position	Promoter type predicted	Conservation in
BjsR1	minus	1,134,665	RpoD	*Bradyrhizobium*
BjsR1	minus	1,134,665	RpoD	*Bradyrhizobium; Rhodopseudomonas*
BjsR2	minus	2,327,788	RpoD, RpoN	*Bradyrhizobium*
BjsR3	minus	3,384,003	RpoD	*Rhodobacterales*
BjsR4	minus	4,718,720	RpoD	*Bradyrhizobium*
BjsR5a,b*	a: plus; b: minus	a: 6,326,856; b: 5,605,382^2^	a: RpoN; b: -	*Bradyrhizobium*
BjsR6a,b,c*	a: minus; b: plus; c: plus	a: 8,446,941; b: 3,129,018; c: 5,115,800	a: RpoN; b: -; c: RpoD	*Bradyrhizobium*
BjsR7a	plus	8,455,582	RpoD	*Bradyrhizobium*
BjsR7b	plus	5,736,937	RpoD	*Bradyrhizobium*
BjsR8	plus	1,929,431**	-	*Bradyrhizobium*
BjsR9	plus	1,779,519	RpoD	*Bradyrhizobium*
BjsR10a,b,c***	minus	a: 1,797,556; b^§^: 1,780,841, 1,780,969; c: no expression	a: -; b: -; c: -	*Bradyrhizobium*

Strand, TSS or 5’-end coordinate (genome position) and predicted promoters are according to ref. 15.

* no discrimination between homologs in the Northern blot hybridization

** processed 5’-end (S12 Fig)

*** only BjsR10a was hybridized

^§^ two convergent TSSs corresponding to complementary sRNAs were detected

–, no predicted promoter upstream of the TSS or 5’-end

We detected sRNA candidates BjsR1 through BjsR7 in free-living conditions and in nodules, and some of them showed clear differences in abundance (BjsR3) or hybridization pattern (BjsR1, BjsR5, and BjsR6) under different conditions (Fig 5A). Since specific probes were not used to discriminate between the several homologs of BjsR5 and BjsR6, in these cases the changes in the hybridization patterns may indicate differential expression of individual homologs (Fig 5A). BjsR8, BjsR9, and BjsR10 were detected exclusively in nodules (Fig 5B), in agreement with the dRNA-seq data (S11 and S12 Figs).

In addition to these new sRNAs we show hybridization results for three non-coding small transcripts with previously validated TSSs [15], which are associated with mRNAs: asRNA AsR1-blr185, sRNA 3’-seR-ntrC, and asRNA 3’-asR-ntrC (Fig 5C; for the distribution of corresponding cDNA reads see S15 Fig). The transcripts sRNA 3’-seR-ntrC and asRNA 3’-asR-ntrC overlap the 3’-UTR of the regulator of nitrogen metabolism *ntrC* [54] in the sense and antisense direction, respectively (S15 Fig). The Northern blot hybridization with a 3’-asRNA-ntrC-specific probe revealed a strong increase in the level of this asRNA in the stationary phase when compared to the exponential growth phase (Fig 5C). In contrast, no signals were obtained in the stationary growth phase with a 3’-seR-ntrC-specific probe (Fig 5C), indicating that the level of this sRNA had been decreased. These results suggest that the asRNA 3’-asR-ntrC may be involved in the down-regulation of 3’-seR-ntrC and/or the upstream *ntrC* operon in the stationary phase. Growth-stage dependent roles of these 3’-UTR associated sRNAs are supported by the previously shown increase in the activity of their promoters in the stationary growth phase compared to the exponential phase [15].

In summary, we identified and characterized orphan sRNAs belonging to ten new RNA families and validated three sRNAs with previously verified TSSs.

### The sRNA BjsR4 is down-regulated under iron stress

**Fig 6:**
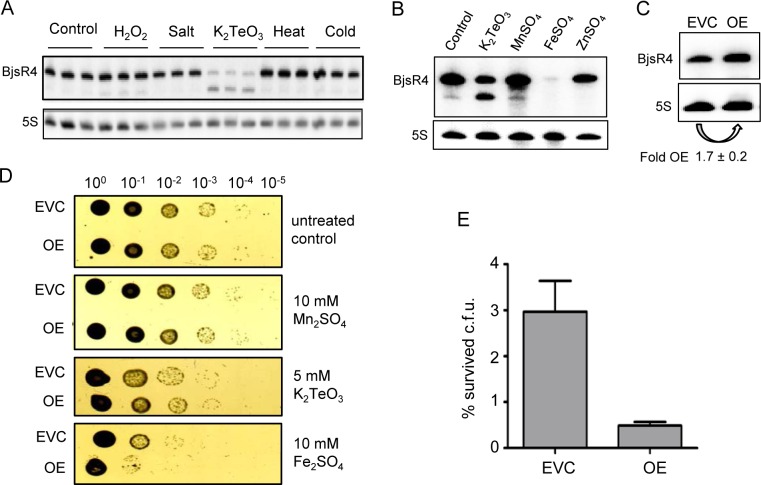
Down-regulation of BjsR4 under tellurite and iron stresses, and impact of this down-regulation on survival at high iron concentration. **A)** The steady-state level of BjsR4 is decreased upon addition of tellurite. Total RNA of three independent cultures was isolated before (control) and after the following stresses: 5 min exposure to 2 mM H_2_O_2_ or 30 min exposure to 50 mM NaCl (salt), 1 mM tellurite (K_2_TeO_2_), 39°C (heat) or 20°C (cold). After hybridization with the BjsR4-specific probe (top panel) the membrane was re-hybridized with the probe specific for 5S rRNA as loading control (bottom panel). **B)** The level of BjsR4 is decreased upon treatment with 1 mM tellurite and 10 mM Fe_2_SO_4_ but not 5 mM Zn_2_SO_4_ or 5mM Mn_2_SO_4_. Cultures were exposed for 30 min to the indicated metal ion solutions and then Northern blot analysis of total RNA was performed with probes specific to BjsR4 and 5S rRNA. **C)** Overexpression of the sRNA BjsR4 in *B*. *japonicum*. A representative Northern blot hybridization of the empty vector control (EVC) and the overexpressing strain (OE) is shown. The detected RNAs are indicated. The fold increase in the BjsR4 amount (Fold OE) given below the panel was calculated from four independent biological replicates. **D)** Increased sensitivity of the BjsR4 overexpressing strain to iron. Cells of the EVC and OE strains were grown to an OD_600_ of 0.2 and 10 mM Mn_2_SO_4,_ 5 mM K_2_TeO_2_ or 10 mM Fe_2_SO_4_ was added. Two hours later the cells were washed, serially diluted, spotted onto PSY plates with spectinomycine and tetracycline and grown for seven days at 30°C Shown is a representative example of six biologically independent experiments with similar results. **E)** Quantitative analysis of percentage colony forming units (c.f.u.) of the EVC and OE strains that survived after exposure to 10 mM Fe_2_SO_4_. Shown are the means and standard deviations from six biologically independent replicates.

We examined the expression of the highly abundant *Bradyrhizobium*-specific sRNA BjsR4 under different stress conditions and found a strong decrease in its steady state level and increased amounts of a degradation product upon addition of tellurite (Fig 6A). To test whether BjsR4 level is changed upon exposure to other metals, Fe_2_SO_4_, Zn_2_SO_4_ and Mn_2_SO_4_ were also added to *B*. *japonicum* cultures. Fig 6B shows that BjsR4 disappeared upon treatment with iron, while zinc and manganese had no effect. This result suggests that down-regulation of BjsR4 could be important for survival of *B*. *japonicum* under iron stress.

To test this, the sequence corresponding to BjsR4 was overexpressed in *B*. *japonicum* under the control of a constitutive promoter using the chromosome-integrated plasmid pRJPaph-bjsr4 (see Methods). As determined by Northern blot hybridization, the BjsR4 amount in the overexpressing (OE) strain was increased only 1.7-fold in comparison to the corresponding empty vector control (EVC) with the integrated plasmid pRJaph-MCS, but this increase was statistically significant (Fig 6C). The OE strain and the EVC were treated with 5 mM tellurite, 10 mM iron, or 10 mM manganese and survival was compared as described previously by others [55]. No differences were observed between the two strains upon treatment with tellurite and manganese (Fig 6D). In contrast, the BjsR4 overexpressing strain was more sensitive to iron than the EVC (Fig 6D and 6E). These results clearly show the impact of down-regulation of BjsR4 under iron stress.

Since high iron concentrations lead to H_2_O_2_ stress [55] and 2 mM H_2_O_2_ did not influence the BjsR4 level (Fig 6A), we performed survival assays using 3 mM H_2_O_2_ and 5 mM H_2_O_2_ to test whether BjsR4 overexpression influences survival under oxidative stress. However, after treatment with H_2_O_2_ the cells did not grow on the plates (not shown).

Fig 6 suggests that BjsR4 controls expression of genes needed for response to tellurite and iron stresses. However, target prediction with TargetRNA2 [56] did not reveal a suitable mRNA target which could explain our observations (S16 Fig) and thus the BjsR4 mechanism remains to be elucidated. In summary, our data suggest that down-regulation of BjsR4 may contribute to survival under high concentration of iron.

### Potential toxin genes in the symbiotic island

**Fig 7:**
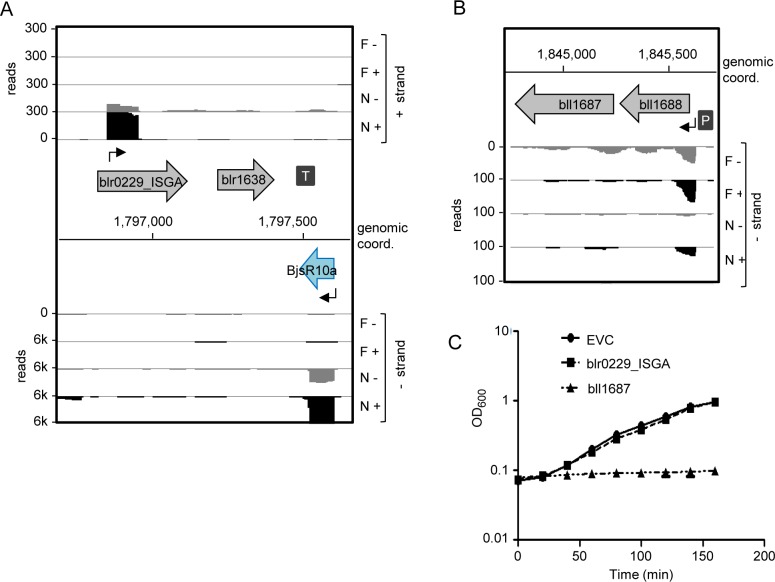
Analysis of two putative toxin genes in the symbiotic island. **A)** cDNA reads at the blr0229_ISGA (*higB*), blr1638 (*higA*) locus. **B)** cDNA reads at the bll1687 (*yhaV*), blr1688 (*prlF*) locus. RNA was isolated from exponentially growing, free-living cells (F) in liquid cultures and from nodules (N). RNA samples were treated (+) or not treated (–) with terminal exonuclease (TEX), which degrades 5’-monophosphorylated (processed) transcripts. The scale of each library is indicated (reads). Grey arrows with indicated gene designations, annotated transcripts; Blue arrows, non-annotated transcript; flexed thin black arrow, mapped TSS; dark grey boxes with P and T, mapped putative promoter and terminator, respectively [15]. **C)** Growth curves of *E*. *coli* containing the empty vector pBAD_Cm (empty vector control, EVC), pBAD_Cm::blr0229_ISGA (blr0229_ISGA) or pBAD::_Cm::bll1687 (bll1687) after induction with arabinose.

By inspection of the BjsR10a locus we found that this sRNA may be complementary to the 3’-UTR of blr1638 (Fig 7A), which encodes a protein with similarity to the bacterial HigA antitoxin [57]. Blr1638 is preceded by a putative ORF encoding a HigB toxin homolog (a newly annotated gene blr0229_ISGA; Fig 6A). This potential TA locus was predicted previously as one of four putative *higBA* loci in *B*. *japonicum* USDA 110 [58,59]. However, a TSS with a high peak height indicating strong transcription in symbiosis is located in blr0229_ISGA (Fig 7A). This questions the functionality of ORF blr0229_ISGA and thus we decided to test its toxic potential by induced expression in *E*. *coli*. As a control we tested in parallel another putative TA system, which was also predicted previously with an even higher confidence [59] and consists of bll1687 and bll1688 encoding YhaV and PrlF homologs, respectively (Fig 7B; [60]). Putative toxin ORFs blr0299_ISGA and bll1687 were cloned into the arabinose-inducible pBAD_Cm vector [48]. Induction of blr0229_ISGA did not show any effect, while bll1687 induction had a clear bacteriostatic effect on *E*. *coli* (Fig 7C).

Thus, we experimentally confirmed the toxic potential of bll1687 but the function of the blr0229_ISGA/blr1638 locus remains elusive. Despite no effect in the heterologous host *E*. *coli*, it cannot be excluded that blr0229_ISGA expression is toxic in *B*. *japonicum*. However, the lack of a detectable TSS upstream and the presence of a strong TSS in blr0229_ISGA suggest that this ORF may have lost its function. Since it is known that a protein antitoxin can repress transcription of the corresponding toxin-antitoxin gene pair [61], it is tempting to speculate that *hig*A homolog blr1638 may encode a transcription regulator needed in symbiosis. Alternatively, it may encode an orphan HigA antitoxin interacting with HigB toxins encoded elsewhere in the genome [58]. We further speculate that BjsR10 may regulate the blr1638 expression by binding to its 3’-UTR.

### Two new REP elements in 3’-UTRs

Recently observed clustering of TSSs in the sense and antisense direction near the ORF ends [15] suggests that sRNAs overlapping with 3’-UTRs of genes and complementary asRNAs are common in *B*. *japonicum* USDA 110. We found that some of the sRNA candidates associated with 3’-UTRs contain new repetitive elements (REPs).

**Fig 8:**
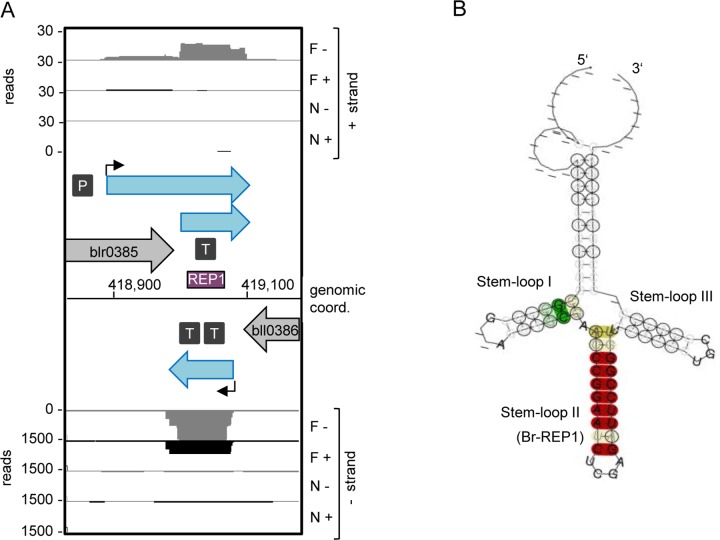
New repetitive element Br-REP1. **A)** cDNA reads at the blr0385, bll0386 locus. Purple box, repetitive element. For other descriptions see the legend to Fig 7. **B)** Predicted secondary structure of Br-REP1 and flanking palindromes. The corresponding alignment is shown in S17 Fig. For the color code see [42].

The first new REP was found in the overlapping 3’-UTRs of the genes blr0385 and bll0386, which are in the convergent orientation. The dRNA-seq data suggested the existence of complementary sRNAs corresponding to this genomic region (Fig 8A). Analysis of their sequences revealed the presence of a repetitive element containing three palindromes, which correspond to three stem-loops at the level of RNA (stem-loops I, II and III in Fig 8B; for the corresponding alignment see S17 Fig). We found that the central stem-loop II with the consensus sequence 5’-CCGGAATCTCGAGATTCCGG-3’ (Br-REP1) is present at approximately 200 sites in intergenic regions (IGRs) in the *B*. *japonicum* USDA 110 genome. Alone or with its flanking palindromes, Br-Rep1 is found in many *Bradyrhizobium* and *Rhodopseudomonas* species, but its abundance differs strongly. For example, in some bradyrhizobia, such as *B*. *elkanii* WSM1741, and also in *Rhodopseudomonas* species, Br-REP1 was found in one to five copies per genome, while one of the flanking palindromes was found in many copies. This suggests fast evolution of repetitive elements in related alphaproteobacteria.

**Fig 9:**
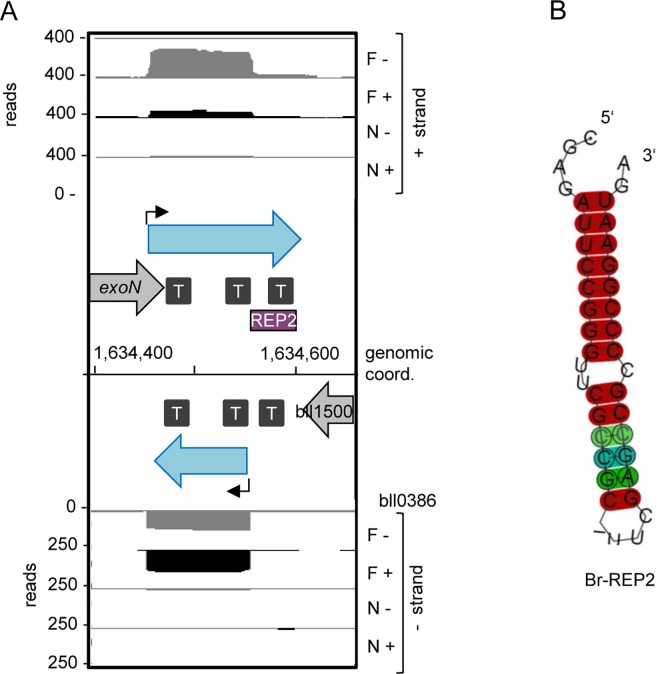
New repetitive element Br-REP2. **A)** cDNA reads at the *exoN*, bll1500 locus. **B)** Predicted secondary structure of Br-REP2. The corresponding alignment is shown in S17 Fig. For other descriptions see the legend to Fig 8.

The second repetitive element with the consensus sequence 5’-CGAG**ATTCCGGGTTCG****GCTCTT-CGAGCCGCCCCGGAAT**GA-3 is present in at least 50 IGRs and the underlined 15 nt in more than 100 additional IGRs in *B*. *japonicum* USDA 110. Its central part (highlighted in bold above) is an imperfect palindrome which we named Br-REP2. The predicted consensus secondary RNA structure of Br-REP2 is shown in Fig 9B and the corresponding alignment in S17 Fig. We found this repetitive element in the IGR between blr1499 *(exoN*, a gene important for symbiosis and involved in exopolysaccharide synthesis, [62]) and bll1500 (Fig 9A). According to our BLASTN analysis, Br-REP2 is widespread in *Bradyrhzobium* and *Rhodopseudominas*. It is a part of a larger repetitive element, which is present in a less conserved form also in other *Bradyrhizobaceae* members like *Nitrobacter*, *Oligotropha*, and *Afipia* (data not shown).

The identification of REPs in *Bradyrhizobium* is important for several reasons. At the DNA level, REP elements in other bacteria were shown to interact with DNA-polymerase I, IHF and gyrase (summarized in ref. 35), and to be targets for insertion sequence elements [63]. Further, as mentioned in the introduction, at the level of RNA REPs can influence transcription termination or mRNA longevity [33,34]. Thus, REPs probably participate in the genome plasticity and gene regulation in *Bradyrhizobium*, but this was not analyzed so far since REP elements were not described for *Bradyrhizobium*. Based on our observations, it is tempting to speculate that REP-containing sRNAs or asRNAs may act in *trans* and influence mRNAs with REPs in their 3’-UTRs. Last but not least, in applied and environmental microbiology primers corresponding to repetitive elements are used for PCR-fingerprinting, which is important for the identification of released bradyrhizobial inoculant strains [36–38]. The REPs described here are inherent parts of bradyrhizobial genomes and could be useful for design of new primers for PCR-fingerprinting of bradyrhizobial strains.

### Transcription interference in a gene active in symbiosis

**Fig 10:**
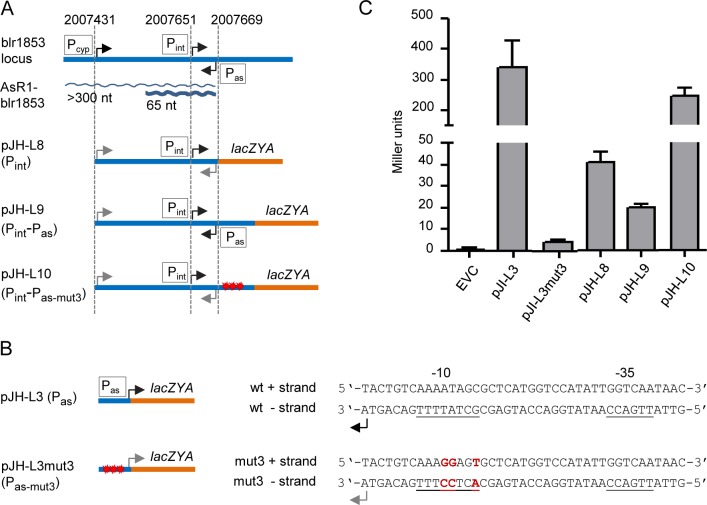
Transcription interference in blr1853. **A)** Blr1853 locus and the structure of *lacZYA* reporter fusions used to analyze transcription interference in blr1853. Plasmid names are indicated. Blue straight line, DNA of the blr1853 locus; blue wave lines, asRNA AsR1-blr1853 (an abundant 65 nt form detected in Fig 5C and a long form previously detected by RT-PCR, [15]); orange line, *lacZYA* genes; thin black flexed arrows, active TSSs; thin gray flexed arrows, inactive TSSs; open boxes with promoter designations, promoters upstream of the TSSs; P_cyp_, blr1853 promoter; P_int_, internal promoter in the sense direction; P_as_, internal promoter in the antisense direction. The genomic coordinates of the TSSs are given on top [15]. Red stars indicate three mutations introduced in P_as_ (see B). The drawing is not to scale. **B)** Reporter fusions used to measure the activity of the wild type (wt) P_as_ and its mutated version P_as-mut3_. Shown are parts of the cloned 63 nt sequence. The TSS of asRNA AsR1-blr1853 is indicated along with the –10 and –15 boxes of the P_as_ promoter. The mutated bases are in red. For other descriptions, see A). **C)** Beta-galactosidase activities of *B*. *japonicum* cells harboring the reporter constructs shown in A) and B). Measurements were performed with aerobic exponentially growing cultures. Shown are the results from three independent experiments with technical duplicates. Error bars indicate the standard deviation.

The asRNA AsR1-blr185 (Fig 5C) is complementary to blr1853 encoding a cytochrome P450 (CYP) (S15 Fig). In addition to their TSSs and corresponding promoters (TSS of blr1853 preceded by P_cyp_ and TSS of AsR1-blr185 preceded by P_as_), a third, internal TSS (iTSS) with an own promoter (P_int_) exists in this locus (Fig 10A and S15 Fig and ref. [15]). This prompted us to consider the possibility that transcription interference may occur between iTSS and aTSS, which are in convergent orientation (Fig 10A). Indeed, the short distance of 17 nt between the two TSSs strongly suggests that formation of an open complex between RNA polymerase and the promoter upstream of the aTSS (P_as_) would exclude formation of an open complex at the promoter upstream of the iTSS (P_int_) [19,64].

To test experimentally whether transcription from P_as_ negatively influences transcription from P_int_, suitable *lac*-reporter plasmids were constructed (Fig 10 A). The plasmid pJH-L8 contains the DNA region from the TSS of blr1853 to the TSS of the asRNA cloned upstream of a promoter-less *lac* operon. In this case the beta-galactosidase activity should rely on P_int_ only. In the second construct, P_as_ was also included, resulting in the plasmid pJH-L9. In line with our expectations, beta-galactosidase activity in cells with pJH-L9 was lower than in cells with pJH-L8 (Fig 10C). However, since the region between P_int_ and the *lac* genes in the two plasmids differs by 63 nt in length and this could account for the difference in activity, we decided to destroy the P_as_ promoter in pJH-L9 by mutagenesis. First we confirmed that exchange of three bases in the -10 region of P_as_ results in a nearly non-functional promoter (Fig 10B and 10C). Then these mutations were introduced in pJH-L9 resulting in pJH-L10. We expected that cells harboring pJH-L10 should have the beta-galactosidase activity similar to that of cells with pJH-L8. However, our measurements revealed a six-fold increase in the beta-galactosidase activity of cells harboring pJH-L10 when compared to those with pJH-L8 (Fig 10C).

This unexpected result suggests that mutations disrupting P_as_ in pJH-L10 had an additional effect on the *lac* operon expression. At one hand, it cannot be excluded that they have resulted in a new promoter in the sense direction, although the mutated sequence of the plus strand does not contain obvious promoter motifs (Fig 10B). Alternatively or in addition, the mutations could affect the half-life of the recombinant *lac* mRNA starting at iTSS in pJH-L10. *B*. *japonicum* harbours a gene encoding RNase E, a key bacterial endoribonuclease RNase E known to cleave in single stranded, AT-rich RNA regions [65]. Thus, the replacement of AT by GG (Fig 10B) in the 5’-UTR of *lacZ* in pJH-L10 could destroy an RNase E cleavage site resulting in enhanced mRNA longevity and thus higher beta-galactosidase activity. The last explanation implies coupling between a half-life determinant (proposed RNase E cleavage site) of blr1853 mRNA and antisense transcription. Thus, at the RNA level the P_as_ sequence could be important for the turnover of blr1853 mRNA and of the overlapping transcript starting at the iTSS, while at the DNA level it may serve to suppress internal transcription by transcription interference.

In summary, Fig 10 shows transcription interference in blr1853. Previously the sequence corresponding to the asRNA AsR1-blr1853 was overproduced in *trans* in the sense and antisense directions without effects on the blr1853 mRNA levels in free-living state and in symbiosis [15]. Therefore, we suggest that the asRNA itself has no function and is a by-product of transcription interference.

## Conclusions

We analyzed the length of 1391 ORFs that were recently added to the re-annotated genome of the soybean symbiont *B*. *japonicum* USDA 110 and found that 1080 of them are sORFs, showing that mostly small genes were missed in the original genome annotation. This finding can be used as a basis for future studies on small proteins in *Bradyrhizobum* and our results on the phylogenetic distribution of these 1080 sORFs open perspectives for further analyses of small proteins in Alphaproteobacteria. In addition, the identification of sRNAs belonging to ten new RNA families will be useful for studies of posttranscriptional gene regulation in *Bradyrhizobium* and related bacteria. The observed down-regulation of the BjsR4 expression upon addition of tellurite and iron may help to understand the mechanisms of survival of bradyrhizobia in polluted soils and to create new strategies of soil bioremediation by rhizobia and their legume hosts [66]. Furthermore, our analyses of two putative TA loci in the symbiotic island confirmed the functionality of the YhaV toxin and suggested that one of the putative genes for the HigB toxin is non-functional. Moreover, we identified new REP elements that probably contribute to genome plasticity and gene regulation, and could be useful for strain identification. Even more, the presented evidence of transcription interference in blr1853 explains the previous failure to detect any effect by massive overexpression of the corresponding asRNA in *trans* [15], a phenomenon also observed in other plant-associated Alphaproteobacteria [67]. In summary, our analyses shed light on poorly investigated genetic elements in *B*. *japonicum* and related bacteria.

## Supporting Information

S1 Fig BLASTN analysis of *B*. *japonicum* sORFs. (PDF)

S2 Fig Blr0566_ISGA gene and protein. (PDF)

S3 Fig Putative blr0250_ISGA gene and protein. (PDF)

S4 Fig Putative bll1319_ISGA gene and protein. (PDF)

S5 Fig cDNA reads, alignment and predicted secondary structure of BjsR1. (PDF)

S6 Fig cDNA reads, alignment and predicted secondary structure of BjsR2. (PDF)

S7 Fig cDNA reads, alignment and predicted secondary structure of BjsR3. (PDF)

S8 Fig cDNA reads and alignment of BjsR4. (PDF)

S9 Fig cDNA reads, alignment and predicted secondary structure of BjsR5 homologs. (PDF)

S10 Fig cDNA reads, alignment and predicted secondary structure of BjsR6 homologs. (PDF)

S11 Fig cDNA reads, alignment and predicted secondary structure of BjsR7 homologs. (PDF)

S12 Fig cDNA reads, alignment and predicted secondary structure of BjsR8. (PDF)

S13 Fig cDNA reads and alignment of BjsR9. (PDF)

S14 Fig cDNA reads, alignment and predicted secondary structure of BjsR10 homologs. (PDF)

S15 Fig cDNA reads for the blr1853 and *ntrC* loci. (PDF)

S16 Fig Predicted BjsR4 targets. (PDF)

S17 Fig Alignment of Br-REP1 and Br-REP2 sequences. (PDF)

S1 Table Plasmids used in this work. (XLSX)

S2 Table Oligonucleotides used in this work. (XLSX)

S3 Table BLASTN analysis of the distribution of homologs of 39 *B*. *japonicum* USDA 110 sORFs with proteomic evidence among bacteria. (XLSX)

S4 Table TBLASTX analysis of the distribution of homologs of 39 *B*. *japonicum* USDA 110 sORFs with proteomic evidence among bacteria. (XLSX)

S5 Table BLASTN analysis of the distribution of homologs of 1080 *B*. *japonicum* USDA 110 sORFs among bacteria. (XLSX)

S6 Table TLASTX analysis of the distribution of homologs of 1080 *B*. *japonicum* USDA 110 sORFs among bacteria. (XLSX)

S7 Table Conservation of 20 sORFs with homologs outside Alphaproteobacteria, which were found by BLASTN. (XLSX)

S8 Table List of organisms with bll1319_ISGA homologs found using BLASTN. (XLSX)

S9 Table Conservation of 47 sORFs with putative homologs outside Alphaproteobacteria, which were found by TBLASTX. (XLSX)

S10 Table Conservation of 21 sORFs that had many homologs in and/or outside Alphaproteobacteria as found by TBLASTX. (XLSX)

S11 Table List of organisms with blr0250_ISGA homologs. (XLSX)
