# Supplementary material for: Small Open Reading Frames, Non-Coding RNAs and Repetitive Elements in Bradyrhizobium japonicum USDA 110
Source: PLoS One. 2016 Oct 27;11(10):e0165429. doi: 10.1371/journal.pone.0165429 (PMC5082802; doi:10.1371/journal.pone.0165429)
Supplement: S1 Fig — (PDF) [file pone.0165429.s001.pdf]

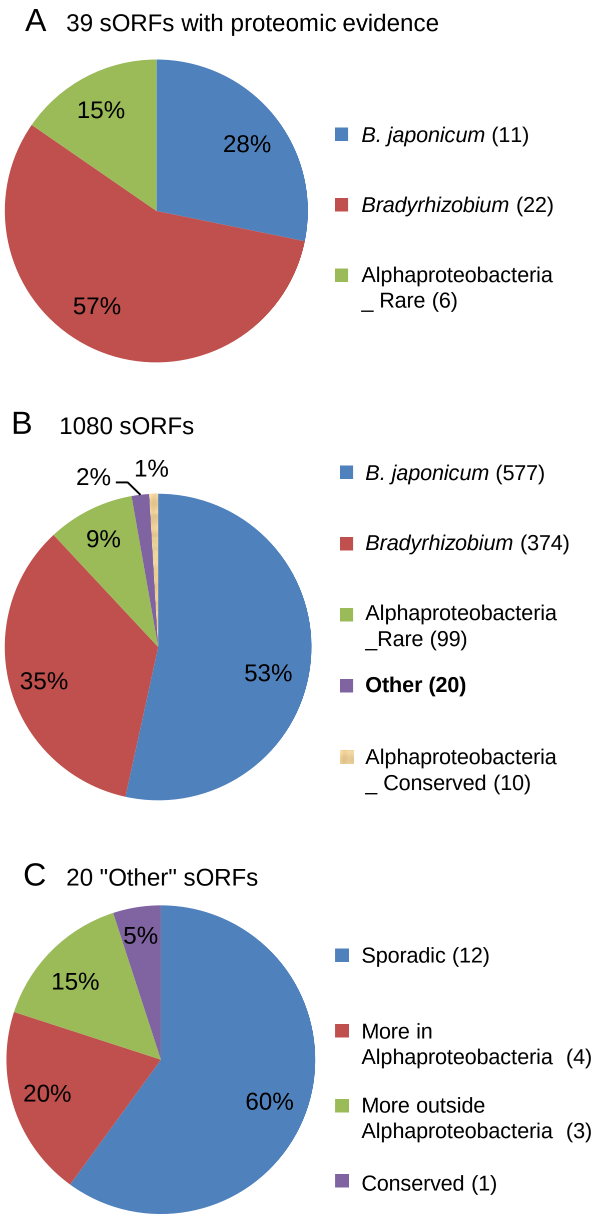

S1 Fig. BLASTN analysis of *B. japonicum* sORFs showing the distribution of homologs among bacteria. **A)** Analysis of 39 sORFs with proteomic evidence. Indicated is their presence only in *B. japonicum* strains (*B. japonicum*), in the genus *Bradyrhizobium* or in Alphaproteobacteria (Table S3). **B)** Analysis of all 1080 sORFs (with and without proteomic evidence). Alphaproteobacteria_Rare – sORFs found in less than five Alphaproteobacteria other than *Bradyrhizobium spp;* Alphaproteobacteria_Conserved – sORFs found in five or more Alphaproteobacteria other than *Bradyrhizobium spp*.; Other – sORFs found in organisms outside Alphaproteobacteria (Table S5). **C)** Analysis of 20 sORFs with homologs outside Alphaproteobacteria (belonging to the category "Other"). Sporadic – sORFs found in less than 10 Alphaproteobacteria and less than 10 organisms outside Alphaproteobacteria; More in Alphaproteobacteria – sORFs found in at least 10 Alphaproteobacteria and less than 10 organisms outside Alphaproteobacteria; More outside Alphaproteobacteria – sORFs found in less than 10 Alphaproteobacteria and at least 10 organisms outside Alphaproteobacteria; Conserved – sORFs found in at least 10 Alphaproteobacteria and at least 10 organisms outside Alphaproteobacteria (Table S7).
