# Supplementary material for: Small Open Reading Frames, Non-Coding RNAs and Repetitive Elements in Bradyrhizobium japonicum USDA 110
Source: PLoS One. 2016 Oct 27;11(10):e0165429. doi: 10.1371/journal.pone.0165429 (PMC5082802; doi:10.1371/journal.pone.0165429)
Supplement: S2 Fig — (PDF) [file pone.0165429.s002.pdf]

A

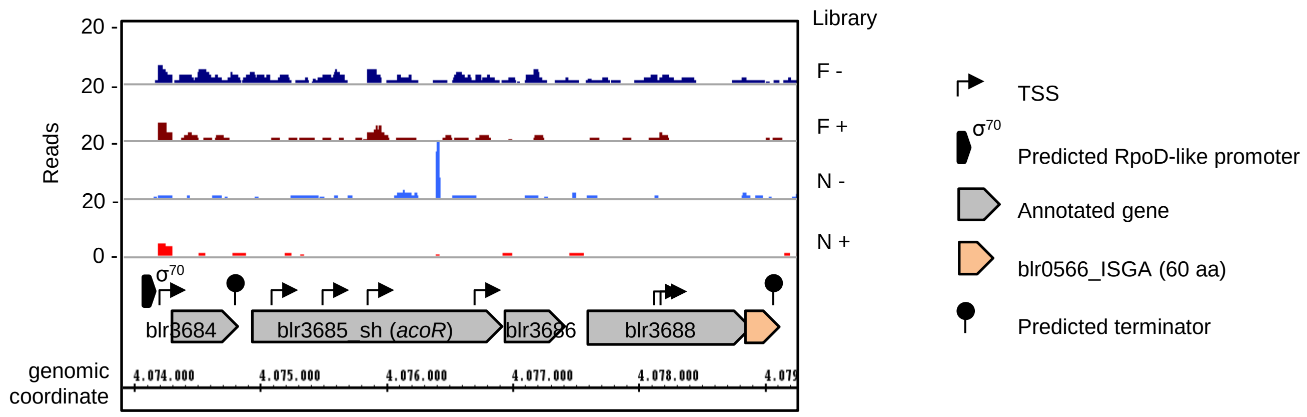

B

```
4078856 cgagcaccag gaggaaatcc atgacagtag cacgattgct aaatgaagcc gaaagtaagg
        atcagttctt caaagagctg gcggaactat cggagcgcat gatcgccgcg cacggaaaag
        agttctcgat gggcgccttg gtgctggcgg cgaggttcat cgtcgaaaat agggcgttcg
        agcgaagcgc agcaacacag tga  4079058
```

C

MTVARLLNEAESKDQFFKELAELSERMIAAHGKEFSMGALVLAARFIVENRAFERSAATQ (60 aa)

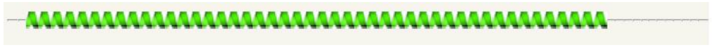

D

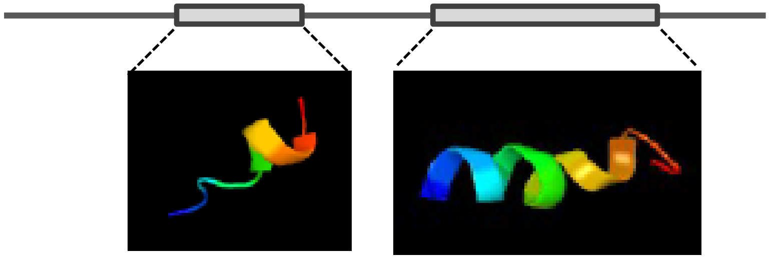

S2 Fig. Blr0566_ISGA gene and protein. A) The blr0566_ISGA gene is weakly expressed at the mRNA level and is probably co-transcribed with upstream genes. Shown are cDNA reads and relevant annotated features (annotated genes, TSSs, promoter and terminators) at the blr0566_ISGA locus [15]. Only the plus strand is shown. The differential RNA-seq (dRNA-seq) was described previously [15]. The analyzed RNA was isolated from exponentially growing, free-living cells (F) in liquid cultures and from nodules (N). RNA samples were treated (+) or not treated (–) with terminal exonuclease (TEX), which degrades 5'-monophosphorylated (processed) transcripts. The scale of each library is indicated (Reads). **B)** Gene sequence with upstream region. The genomic coordinates are indicated, the start and stop codons are in red and the putative Shine-Dalgarno sequence is underlined. **C)** Protein sequence with proposed secondary structure. Blue, peptides detected by proteomics [15]. The peptides were detected only in nodules. **D)** Proposed three-dimensional structure of indicated protein parts . Phyre[2] was used for secondary and tertiary structure modeling [43].
