# Supplementary material for: Small Open Reading Frames, Non-Coding RNAs and Repetitive Elements in Bradyrhizobium japonicum USDA 110
Source: PLoS One. 2016 Oct 27;11(10):e0165429. doi: 10.1371/journal.pone.0165429 (PMC5082802; doi:10.1371/journal.pone.0165429)
Supplement: S3 Fig — (PDF) [file pone.0165429.s003.pdf]

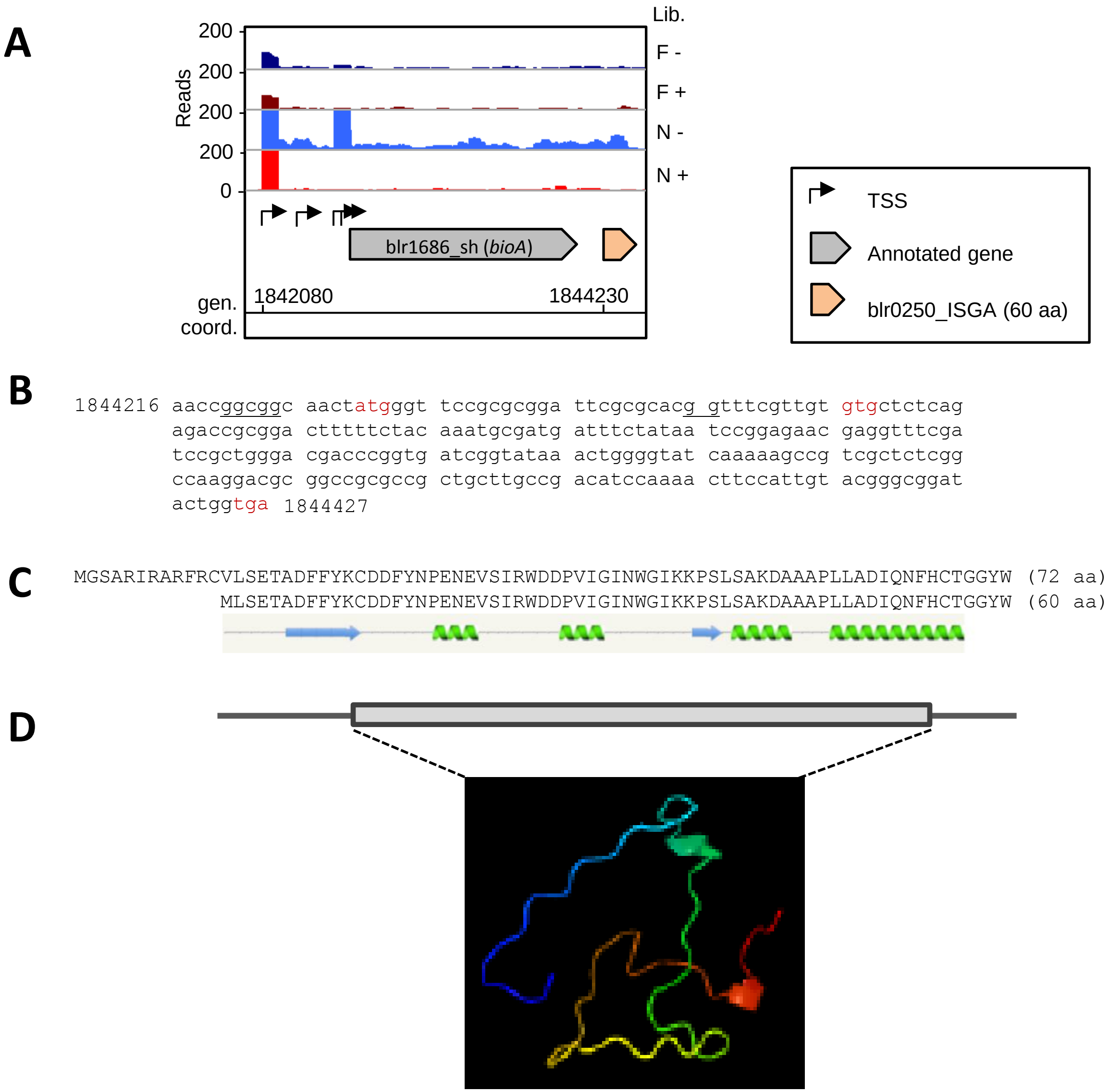

S3 Fig. Putative blr0250_ISGA gene and protein. A) The blr0250_ISGA gene is probably co-transcribed with blr1686_sh [15]. Shown are cDNA reads, annotated genes and an upstream TSSs. Promoters and terminators were not mapped in this genomic region. For further annotated features see ref. [15]. Only the plus strand is shown. The coordinates of the most upstream TSS and of the sORF start are given. The differential RNA-seq (dRNA-seq) was described previously [15]. The analyzed RNA was isolated from exponentially growing, free-living cells (F) in liquid cultures and from nodules (N). RNA samples were treated (+) or not treated (–) with terminal exonuclease (TEX), which degrades 5'-monophosphorylated (processed) transcripts. The scale of each library is indicated (Reads). **B)** Gene sequence with upstream region. Two possible start codons *in frame* were found. The genomic coordinates are indicated, the start and stop codons are in red and the putative Shine-Dalgarno sequences are underlined. **B)** Protein sequence with proposed secondary structure. **C)** Proposed three-dimensional structure of indicated protein parts. Phyre^2 was used for secondary and tertiary structure modeling [43].
