# Supplementary material for: Small Open Reading Frames, Non-Coding RNAs and Repetitive Elements in Bradyrhizobium japonicum USDA 110
Source: PLoS One. 2016 Oct 27;11(10):e0165429. doi: 10.1371/journal.pone.0165429 (PMC5082802; doi:10.1371/journal.pone.0165429)
Supplement: S4 Fig — (PDF) [file pone.0165429.s004.pdf]

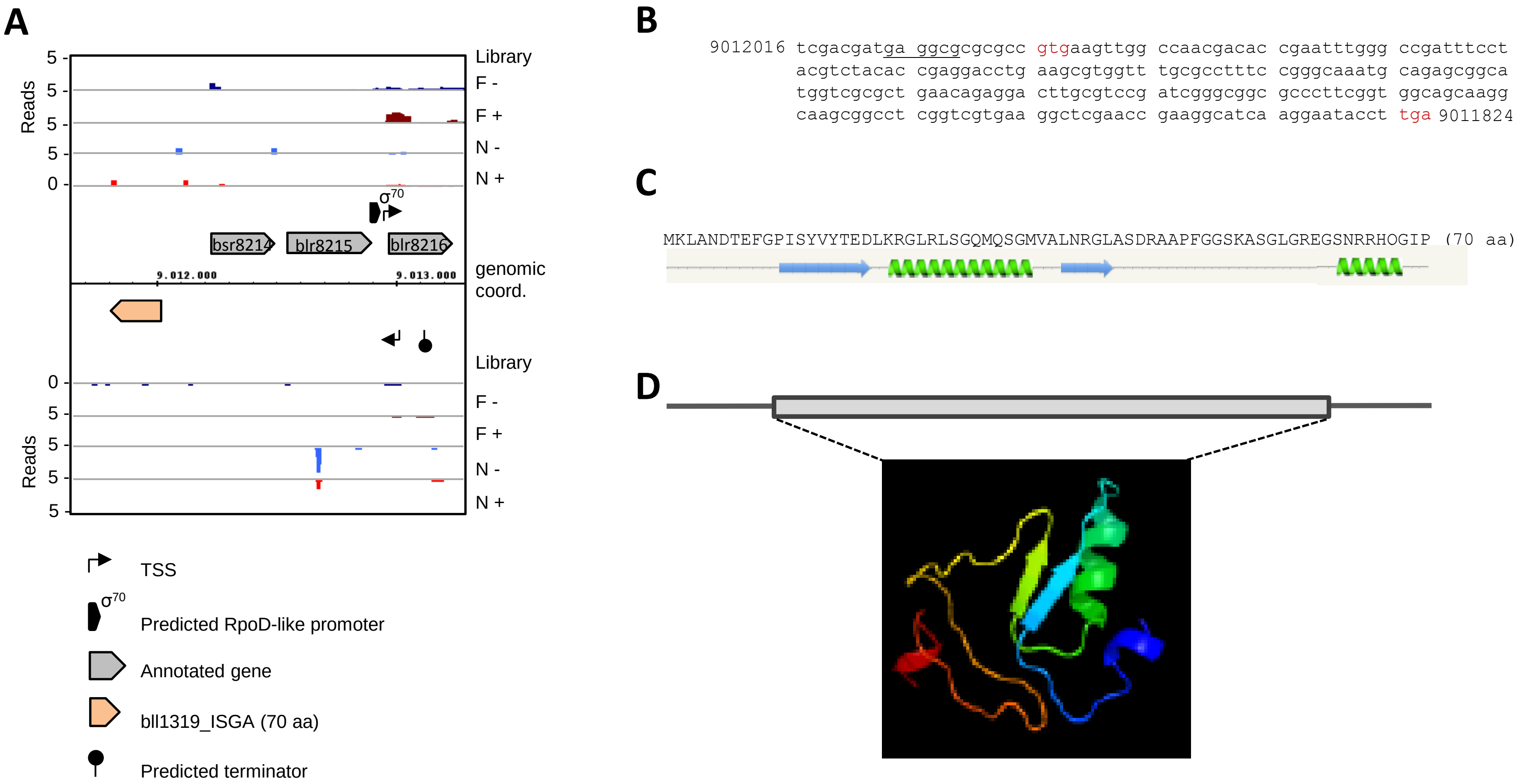

S4 Fig. Putative bll1319_ISGA gene and protein. A) The bll1319_ISGA gene is not expressed at the mRNA level under the applied conditions. Shown are cDNA reads and relevant annotated features (annotated genes, TSSs, promoter and terminators) at the bll1319_ISGA locus [15]. Both strands are shown. The differential RNA-seq (dRNA-seq) was described previously [15]. The analyzed RNA was isolated from exponentially growing, free-living cells (F) in liquid cultures and from nodules (N). RNA samples were treated (+) or not treated (–) with terminal exonuclease (TEX), which degrades 5'-monophosphorylated (processed) transcripts. The scale of each library is indicated (Reads). Genes bsr8214, blr815 and blr816 show homology to transposase genes. Since bll1319_ISGA homologs were found mainly in Alphaproteobacteria and in Actinobacteria, we analyzed the flanking genes in these bacterial groups. No syntheny was found and thus there is no indication for the spread of bll1319_ISGA by horizontal gene transfer. **B)** Gene sequence with upstream region. The genomic coordinates are indicated, the start and stop codons are in red and the putative Shine-Dalgarno sequence is underlined. **B)** Protein sequence with proposed secondary structure. **C)** Proposed three-dimensional structure of indicated protein parts. Phyre^2 was used for secondary and tertiary structure modeling [43].
