# Supplementary material for: Small Open Reading Frames, Non-Coding RNAs and Repetitive Elements in Bradyrhizobium japonicum USDA 110
Source: PLoS One. 2016 Oct 27;11(10):e0165429. doi: 10.1371/journal.pone.0165429 (PMC5082802; doi:10.1371/journal.pone.0165429)
Supplement: S7 Fig — (PDF) [file pone.0165429.s007.pdf]

BjsR3

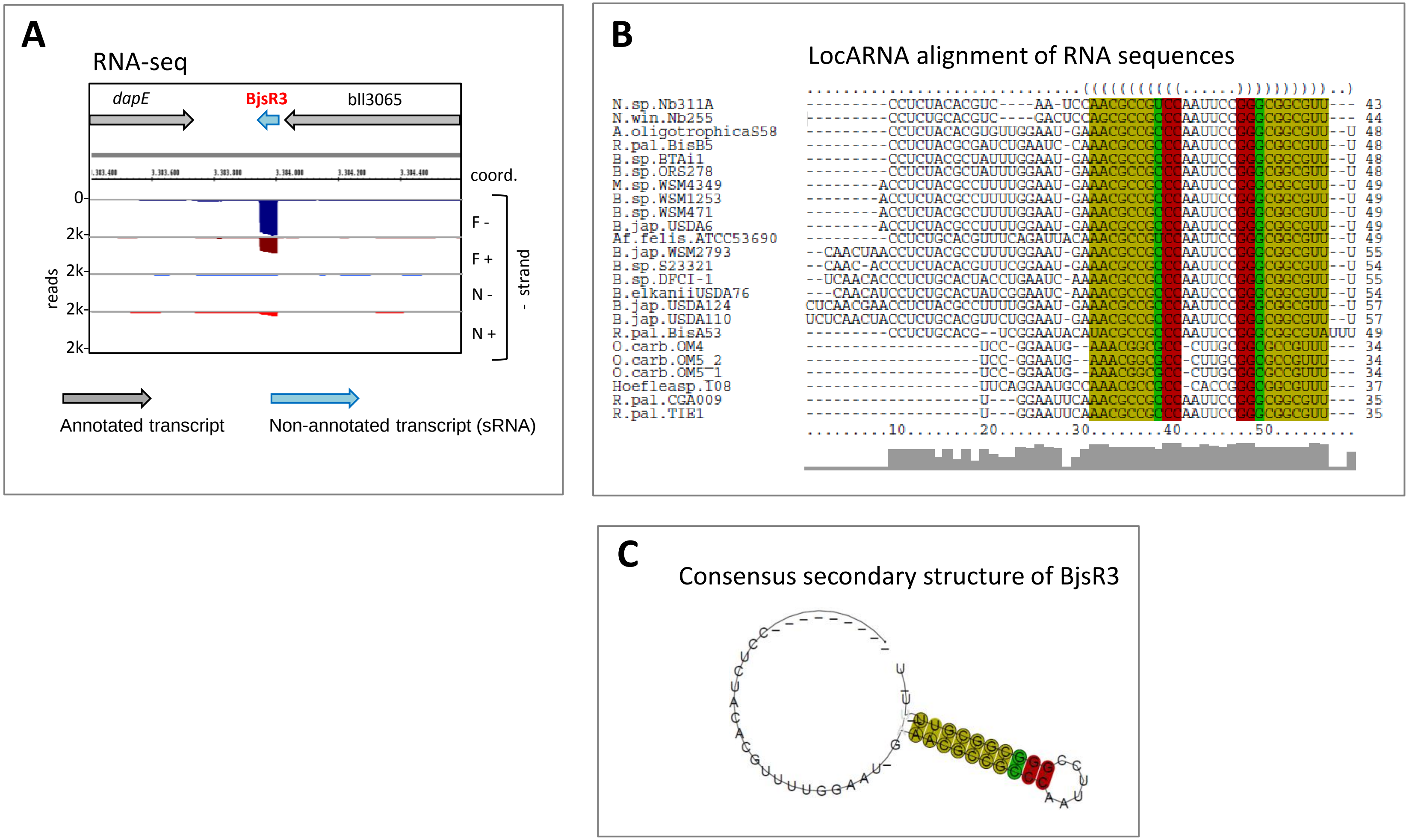

S7 Fig. cDNA reads, alignment and predicted secondary structure of BjsR3 with a TSS at genomic position 3,384,003. A) cDNA reads mapped to the genome. RNA was isolated from exponentially growing, free-living cells (F) in liquid cultures and from nodules (N). RNA samples were treated (+) or not treated (–) with terminal exonuclease TEX. Annotated and non-annotated transcripts are indicated [15] . All libraries were adjusted to the indicated scale (reads). **B)** LocARNA alignment of RNA sequences of *Bradyrhizobiaceae* members. **C)** Consensus secondary structure based on the alignment shown in B). For the color code see ref. [42]. B. jap., *Bradyrhizobium japonicum*; B. sp., *Bradyrhizobium sp.*; B. elkanii, *Bradyrhizobium elkanii*; R. pal., *Rhodopseudomonas palustris*; N.sp., *Nitrobacter*.sp.; N.win., *Nitrobacter winogradskyi*; A. oligotrophica, *Agromonas oligotrophica* (*Bradyrhizobium oligotrophicum*); M.sp., *Mesorhizobium* sp.; Af. felis, *Afipia felis*; O.carb. *Oligotropha carboxidovorans*. In addition, sequences homologous to the conserved terminator stem-loop of BjsR3 are present in many members of *Rhizobiaceae* and *Rhodobacteraceae.*
