# Supplementary material for: Small Open Reading Frames, Non-Coding RNAs and Repetitive Elements in Bradyrhizobium japonicum USDA 110
Source: PLoS One. 2016 Oct 27;11(10):e0165429. doi: 10.1371/journal.pone.0165429 (PMC5082802; doi:10.1371/journal.pone.0165429)
Supplement: S8 Fig — (PDF) [file pone.0165429.s008.pdf]

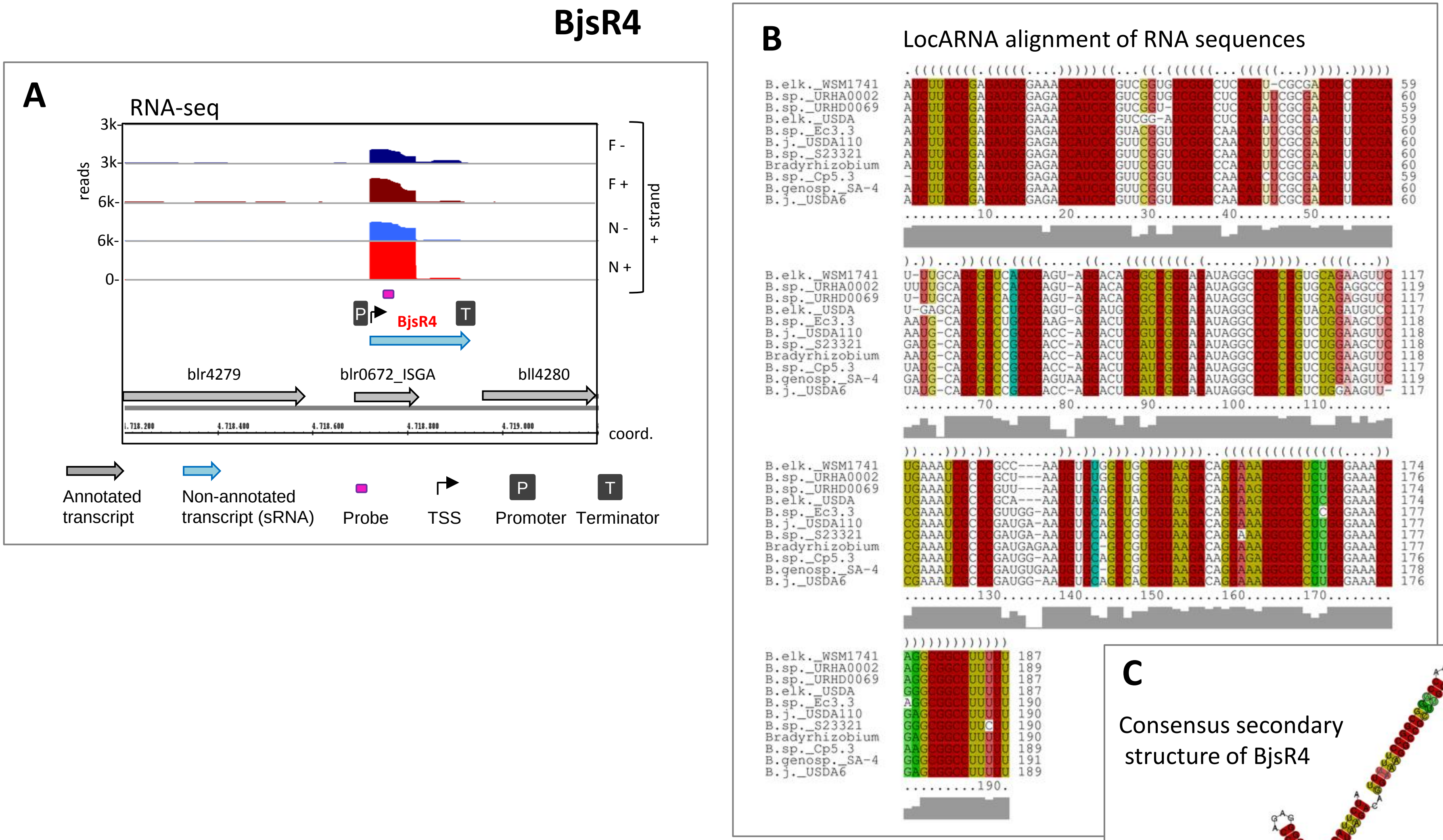


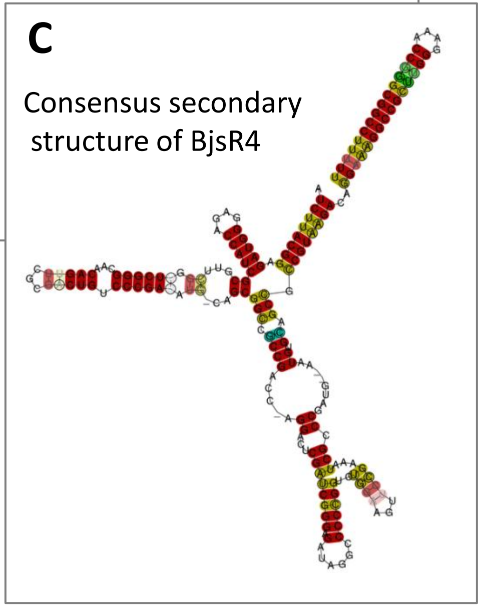


**S8 Fig. cDNA reads and alignment of BjsR4 with a TSS at genomic position 4,718,720. A)** cDNA reads mapped to the genome. RNA was isolated from exponentially growing, free-living cells (F) in liquid cultures and from nodules (N). RNA samples were treated (+) or not treated (–) with terminal exonuclease TEX. Annotated and non-annotated transcripts are indicated [15] . All libraries were adjusted to the indicated scale (reads). **B)** LocARNA alignment of RNA sequences. For the color code see ref. [42]. B.j., *Bradyrhizobium japonicum*; B. sp., *Bradyrhizobium sp.*; B. genosp, *Bradyrhizobium* genosp.; B. elk., *Bradyrhizobium elkanii*.
