# Supplementary material for: Small Open Reading Frames, Non-Coding RNAs and Repetitive Elements in Bradyrhizobium japonicum USDA 110
Source: PLoS One. 2016 Oct 27;11(10):e0165429. doi: 10.1371/journal.pone.0165429 (PMC5082802; doi:10.1371/journal.pone.0165429)
Supplement: S11 Fig — (PDF) [file pone.0165429.s011.pdf]

BjsR7

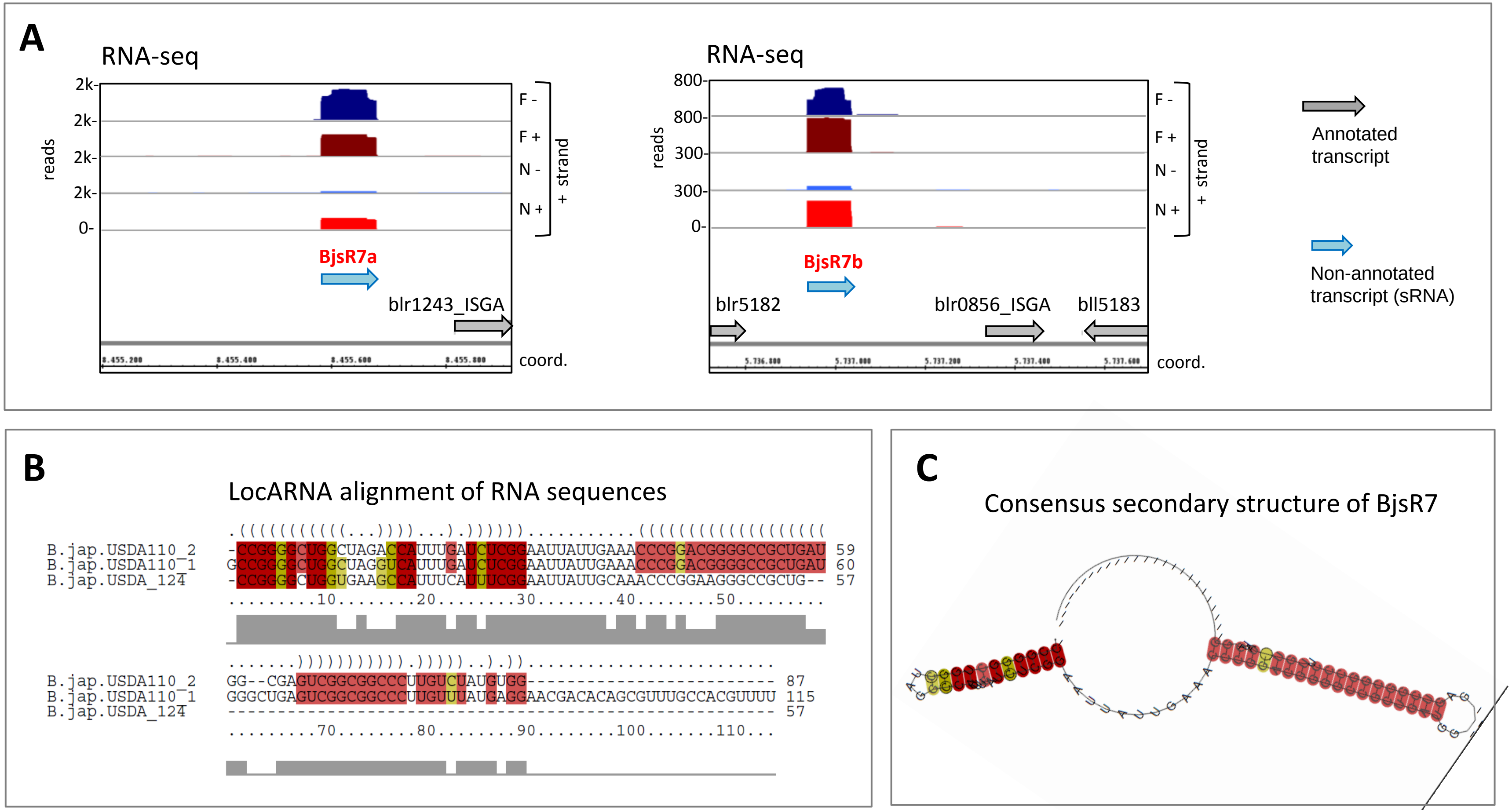

S11 Fig. cDNA reads, alignment and predicted secondary structure of BjsR7 homologs with TSSs at genomic positions 8,455,582 (BjsR7a) and 5,736,937 (BjsR7b). A) cDNA reads mapped to the genome. RNA was isolated from exponentially growing, free-living cells (F) in liquid cultures and from nodules (N). RNA samples were treated (+) or not treated (–) with terminal exonuclease TEX. Annotated and non-annotated transcripts are indicated [15] . All libraries were adjusted to the indicated scale (reads). **B)** LocARNA alignment of RNA sequences. **C)** Consensus secondary structure. For the color code see ref. [42]. B. jap., *Bradyrhizobium japonicum*; B. sp., *Bradyrhizobium sp*.
