# Supplementary material for: Small Open Reading Frames, Non-Coding RNAs and Repetitive Elements in Bradyrhizobium japonicum USDA 110
Source: PLoS One. 2016 Oct 27;11(10):e0165429. doi: 10.1371/journal.pone.0165429 (PMC5082802; doi:10.1371/journal.pone.0165429)
Supplement: S14 Fig — (PDF) [file pone.0165429.s014.pdf]

BjsR10

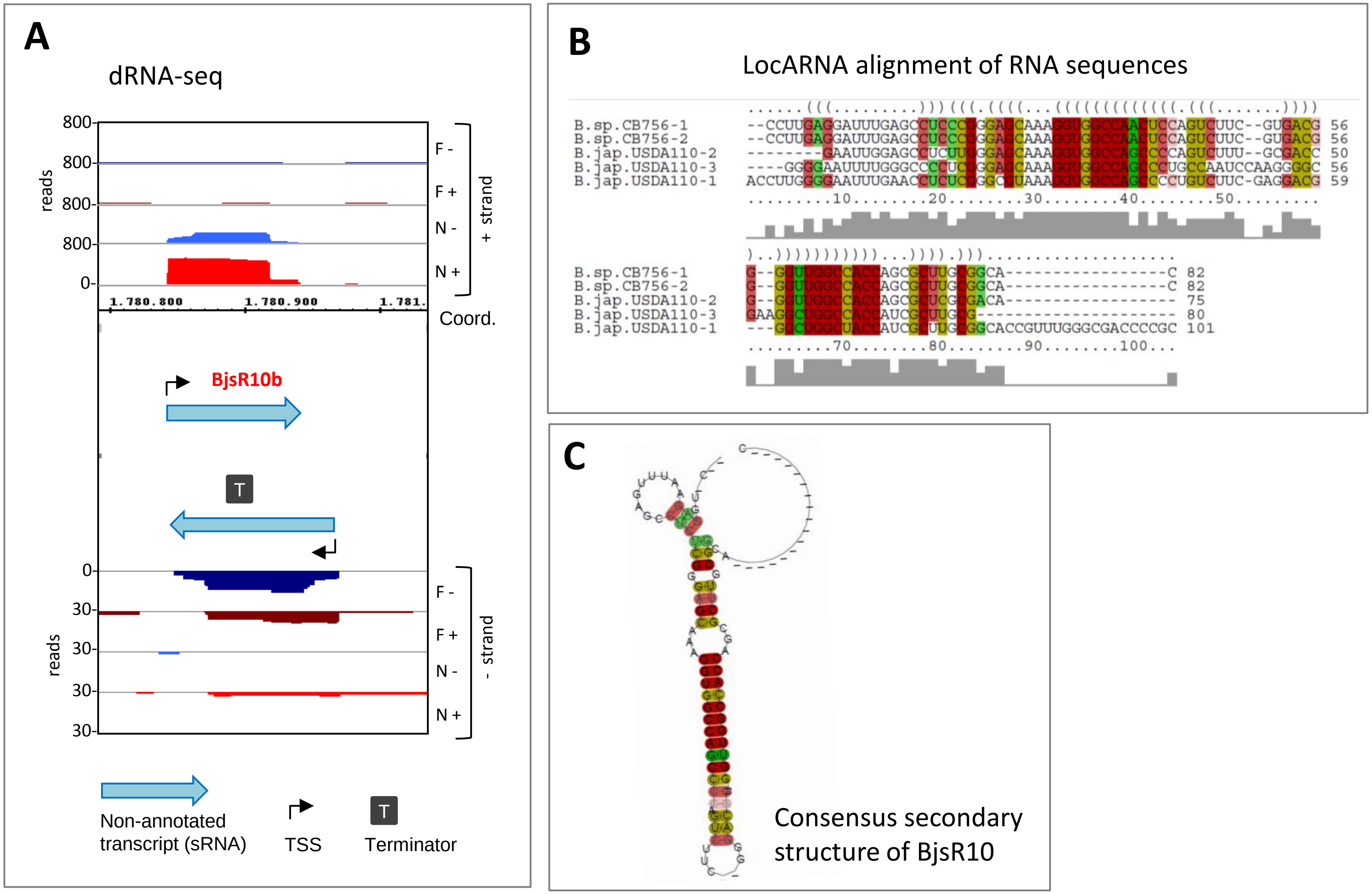

S14 Fig. Alignment and predicted secondary structure of BjsR10 homologs, and cDNA reads of BjsR10b. A) cDNA reads mapped to the BjsR10b locus. Two complementary sRNAs were detected, which are transcribed from convergent TSSs located at 1,780,841 and 1,780,969. The sRNA corresponding to the + strand is expressed only in nodules (similarly to BjsR10a, see Fig. 6 in the main text), while the sRNAs on the opposite strand is expressed predominantly in free-living bacteria. A BjsR10-like sequence is present also between the genomic coordinates 9.040,061 and 9.040,982 (putative BjsR10c locus), but this locus is not expressed under the tested conditions. RNA was isolated from exponentially growing, free-living cells (F) in liquid cultures and from nodules (N). RNA samples were treated (+) or not treated (–) with terminal exonuclease TEX. Annotated and non-annotated transcripts are indicated [15] . All libraries were adjusted to the indicated scale (reads). **B)** LocARNA alignment of RNA sequences. **C)** Consensus secondary structure. For the color code see ref. [42]. B. jap., *Bradyrhizobium japonicum*; B. sp., *Bradyrhizobium sp*.
