# Supplementary material for: Small Open Reading Frames, Non-Coding RNAs and Repetitive Elements in Bradyrhizobium japonicum USDA 110
Source: PLoS One. 2016 Oct 27;11(10):e0165429. doi: 10.1371/journal.pone.0165429 (PMC5082802; doi:10.1371/journal.pone.0165429)
Supplement: S16 Fig — (PDF) [file pone.0165429.s016.pdf]

S16 Fig. Putative targets of BjsR4 predicted by TargetRNA2

```
1        blr3819    metapyrocatechase
Energy: -20.11                   p-value: 0.000

sRNA                    40      ACGGGCUUGGCUU       28
                                 |||||||||||
mRNA  (blr3819)         5       AGCCCGAACCGAU       17
```

```
2        bll5596    ABC transporter substrate-binding protein
Energy: -20.06          p-value: 0.000

sRNA                    40      ACGGGCUUGGCUUGCGC       24
                                 |  |||||||||:||
mRNA  (bll5596)         -65     AGAACGAACCGAAUGCC      -49
```

```
3        cheR1      protein-glutamate O-methyltransferase
Energy: -17.6           p-value: 0.000

sRNA                    94      AGGGCUGGCUCAGGACC       78
                                 |||||||||||  ||
mRNA  (cheR1)           5       CCCCGACCGAGU-AUGA       20
```

```
4        panC       pantoate--beta-alanine ligase
Energy: -16.47          p-value: 0.000

sRNA                    92      GGCUGGCUCAGGAC--CAGC       75
                                ||  |:||||||||  |||
mRNA  (panC)            -15     CCCCCUGAGUCCUGAUGUCA        6
```

```
5        blr8145    two-component hybrid sensor and regulator
Energy: -14.8           p-value: 0.001

sRNA                    38      GGGCUUGGCUUGCGC       24
                                 |:|:|||||||||
mRNA  (blr8145)         -64     GCUGGACCGAACGCA      -50
```

```
6        blr0474    hypothetical protein
Energy: -14.25          p-value: 0.001

sRNA                    44      GACAACGGGCUUGGCUUGCG       25
                                 |||| |||||||:|| |||
mRNA  (blr0474)         -38     AUGUU-CCCGAACUGACCGC      -20
```

```
7        bll0849    serine protease
Energy: -14.2           p-value: 0.001

sRNA                    40      ACGGGCUUGGCUUGC       26
                                 |||| ||||||||
mRNA  (bll0849)         -75     CGCCCCAACCGAACA      -61
```

Only the top seven targets are shown.

Reference:
TargetRNA2: identifying targets of small regulatory RNAs in bacteria. Mary Beth Kery, Monica Feldman, Jonathan Livny, and Brian Tjaden. *Nucleic Acids Research*, 42(W1): W124-W129, 2014.
