# Supplementary material for: Small Open Reading Frames, Non-Coding RNAs and Repetitive Elements in Bradyrhizobium japonicum USDA 110
Source: PLoS One. 2016 Oct 27;11(10):e0165429. doi: 10.1371/journal.pone.0165429 (PMC5082802; doi:10.1371/journal.pone.0165429)
Supplement: S17 Fig — (PDF) [file pone.0165429.s017.pdf]

Br-REP1 and Br-REP2 alignments

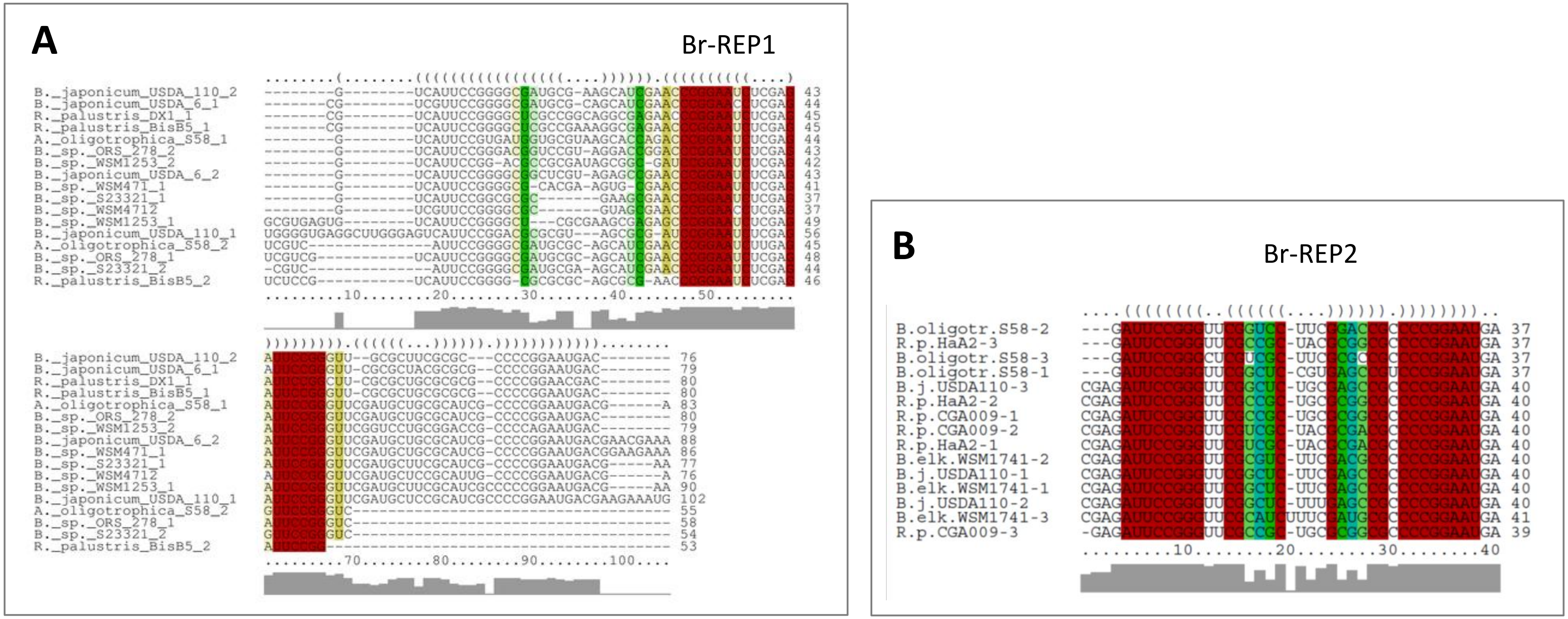

S17 Fig. LocARNA alignments of RNA-sequences containing Bj-REP1 (A) and Bj-REP2 (B). For the color code see ref. [42]. B._japonicum. or B.j., *Bradyrhizobium japonicum*; B._sp., *Bradyrhizobium sp.*; B.elk., *Bradyrhizobium elkanii*; R._palustris or R.p., *Rhodopseudomonas palustris*; A_oligotrophica or B. oligotr., *Agromonas oligotrophica* (*Bradyrhizobium oligotrophicum*); .
