# Supplementary material for: Small Open Reading Frames, Non-Coding RNAs and Repetitive Elements in Bradyrhizobium japonicum USDA 110
Source: PLoS One. 2016 Oct 27;11(10):e0165429. doi: 10.1371/journal.pone.0165429 (PMC5082802; doi:10.1371/journal.pone.0165429)
Supplement: S15 Fig — (PDF) [file pone.0165429.s015.pdf]

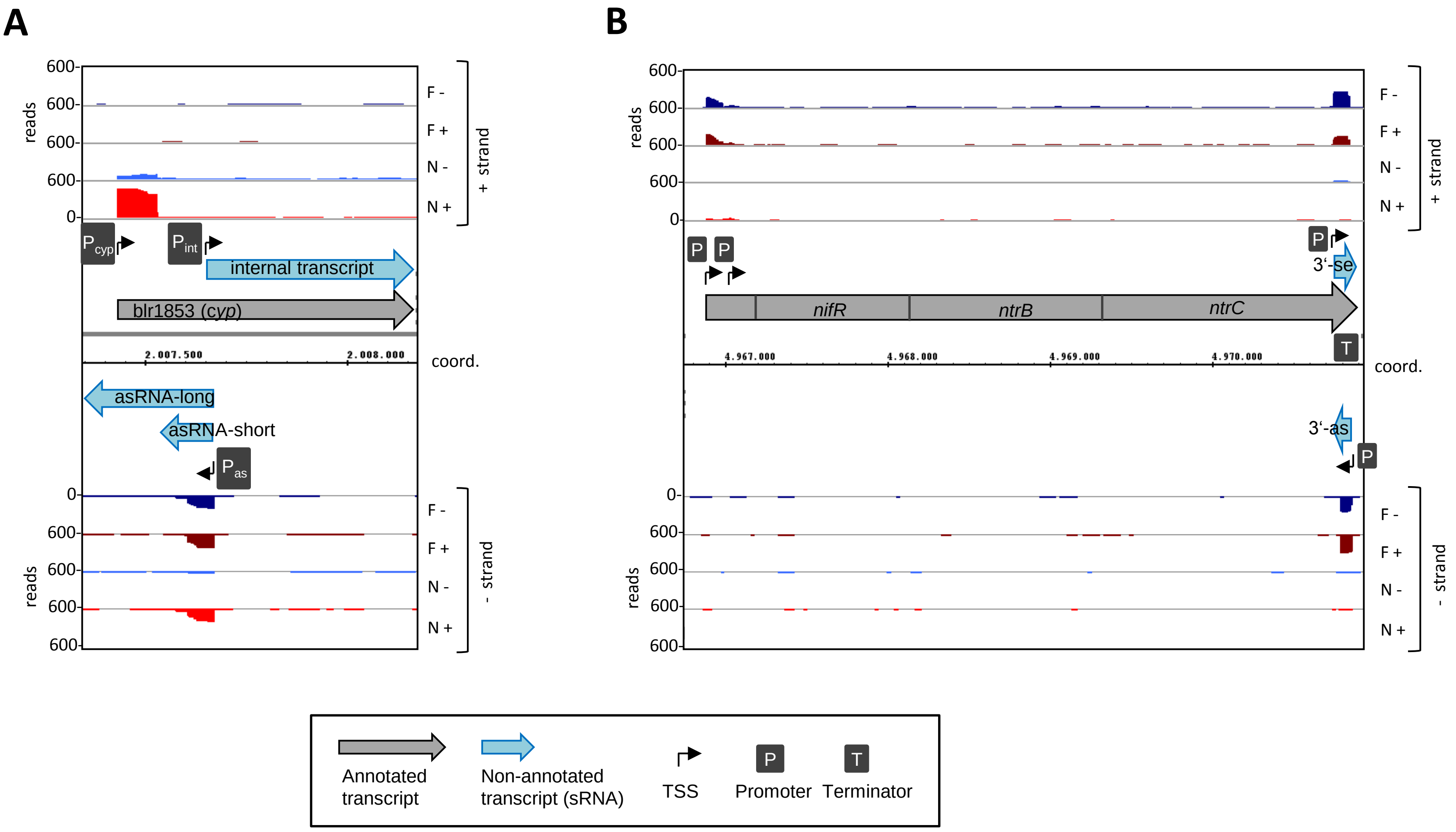

S15 Fig. cDNA reads and relevant annotated features at the blr1853 (A) and *ntrC* (B) loci. All libraries were adjusted to the indicated scale (reads). RNA was isolated from exponentially growing, free-living cells (F) in liquid cultures and from nodules (N). RNA samples were treated (+) or not treated (–) with terminal exonuclease TEX. Annotated and non-annotated transcripts, transcription start sites (TSS), prompters (P) and terminators (T) are indicated according to ref. [15]. **A)** The short form of the asRNA AsR1-blr1853 (asRNA-short) of 65 nt was detected by Northern blot analysis in this study (see Fig. 5C in the main text). The long form was detected previously using RT-PCR [15]. **B)** The sRNAs associated with the 3'-UTR of *ntrC* , 3'-seR-ntrC (3'-se) and 3'-asR-ntrC (3'-as) were validated in this study by Northern blot hybridization (see Fig. 5C in the main text).
